# Endometrial immune dysregulation shapes CD8^+^ T cell mediated reproductive outcomes in recurrent implantation failure: an integrated mechanistic and predictive analysis

**DOI:** 10.3389/fimmu.2026.1788922

**Published:** 2026-03-30

**Authors:** Shan Jiang, Yeqing Fu, Xiufeng Lin, Qingni Li, Zhibin Huang, Cong Zhou, Yecheng Zou, Yutong Li

**Affiliations:** 1 Reproductive Center, Boai Hospital of Zhongshan, Zhongshan, Guangdong, China; 2 The First School of Clinical Medicine, Guangdong Medical University, Zhanjiang, Guangdong, China

**Keywords:** CD8+ T cells, immune disorder, implantation failure number, machine learning, predictive modeling, recurrent implantation failure

## Abstract

**Background:**

The recurrent implantation failure (RIF) remains a major clinical challenge in assisted reproduction. While endometrial immune dysregulation is implicated, its specific role and interaction with clinical factors are poorly defined. The lack of integrated, multimodal predictive models that combine clinical history with immune profiling limits personalized management.

**Methods:**

This study conducted a retrospective cohort study of 110 RIF patients, collecting comprehensive clinical and immune parameters. Traditional statistics and machine learning were employed to identify key predictors and build predictive models. Model interpretability was assessed using SHAP analysis, and causal pathways were explored *via* mediation analysis and restricted cubic splines.

**Results:**

Previous implantation failure number was the strongest negative predictor (aOR = 0.74, 95% CI 0.60–0.91, *P* = 0.004). Endometrial CD8^+^ T cell proportion exhibited a positive, threshold−dependent effect: above 2.0%, each 1% increase raised the odds of success by 25% (aOR = 1.25, 95% CI 1.03–1.52, *P* = 0.025). Embryo quality was an independent positive predictor (aOR = 1.62, 95% CI 1.04–2.53, *P* = 0.033). Machine−learning modeling (XGBoost) achieved an AUC of 0.762 (95% CI 0.734–0.790). Mediation analysis revealed that 22.8% of the total effect of immune dysregulation on outcome was mediated through CD8^+^ T cells. Furthermore, the protective effect of CD8^+^ T cells was significantly enhanced in patients with severe immune disorder (interaction *P* = 0.034).

**Conclusion:**

This integrated clinical-immune signature underscores the pivotal, threshold-dependent role of endometrial CD8^+^ T cells and the cumulative burden of previous failures in RIF. The internally validated machine-learning model offers a prognostic tool, and the elucidated CD8^+^ T cell-mediated pathway suggests a target for immunomodulation, advancing the precision management of RIF.

## Highlights

- Endometrial CD8+ T cell proportion above 2.0% significantly increases pregnancy odds in RIF, showing a clear threshold effect.
- The machine‑learning (XGBoost) model integrating clinical history and immune profiles predicts pregnancy success with AUC 0.762.
- CD8+ T cells mediate 22.8% of the adverse effect of immune dysregulation, revealing a key mechanistic pathway.
- The cumulative number of previous implantation failures is the strongest independent negative predictor of outcome.
- Research provides a data‑driven framework for personalized prognosis and targeted immunotherapy in RIF.

## 1 Introduction

Despite remarkable advances in assisted reproductive technologies (ART) (1), the recurrent implantation failure (RIF) remains a profound clinical dilemma (2, 3), affecting a significant proportion of couples seeking fertility treatment (4). Traditional paradigm has predominantly focused on embryonic factors, such as genetic integrity and morphology (5, 6). However, the consistent success achieved with the euploid embryo transfers underscores a critical, often overlooked determinant: the endometrial microenvironment. Within this milieu, the immune homeostasis is postulated to be a cornerstone of endometrial receptivity (7, 8), governing the delicate dialogue between the semi-allogeneic embryo and the maternal endometrium (9, 10). Dysregulation of this immune network is increasingly implicated in the pathophysiology of RIF, transforming the endometrium from a receptive host into a hostile environment (11, 12).

Current understanding of endometrial immunity in RIF, however, remains fragmented. Studies have often investigated isolated immune cell populations—such as natural killer (NK) cells or macrophages—yielding the inconsistent and sometimes contradictory results (13–15). This siloed approach fails to capture systemic, multifactorial nature of immune dysregulation. Moreover, a critical gap exists in integrating these immune signatures with the robust clinical histories, particularly the cumulative burden of past failures, which may reflect an entrenched pathological microenvironment (16, 17). Consequently, there is a pressing need for a unified framework that synthesizes multidimensional clinical and immunological data to move beyond descriptive associations toward predictive and mechanistic insights.

Emergence of the advanced analytics and machine learning (ML) offers a transformative opportunity to address this complexity (18, 19). Methodologies excel at identifying subtle, non-linear patterns and interactions within the high-dimensional datasets that elude conventional statistics (20, 21). Their application in the reproductive medicine, however, has been limited, seldom used to integrate clinical narrative of repeated failure with deep immune phenotyping to predict individual outcomes and elucidate causal pathways (22, 23). We hypothesize that RIF is characterized by a distinct, integrated clinical-immune signature, where the specific immune aberrations interact with the cumulative history of implantation failure to determine reproductive outcomes.

To test this hypothesis, we conducted a systematic study of 110 RIF patients by integrating detailed clinical profiles with comprehensive endometrial immune mapping. Employing an analytical strategy that combined causal inference with machine learning, we aimed to identify key predictive variables, build a robust prognostic model, and elucidate mechanistic pathways, particularly threshold effects and mediation relationships. This work establishes a data−driven framework for RIF and, more importantly, reveals pivotal and targetable role of endometrial CD8^+^ T cells, pointing toward precise prognosis and mechanism−informed immunotherapy.

## 2 Materials and methods

### 2.1 Research design

**Figure 1:**
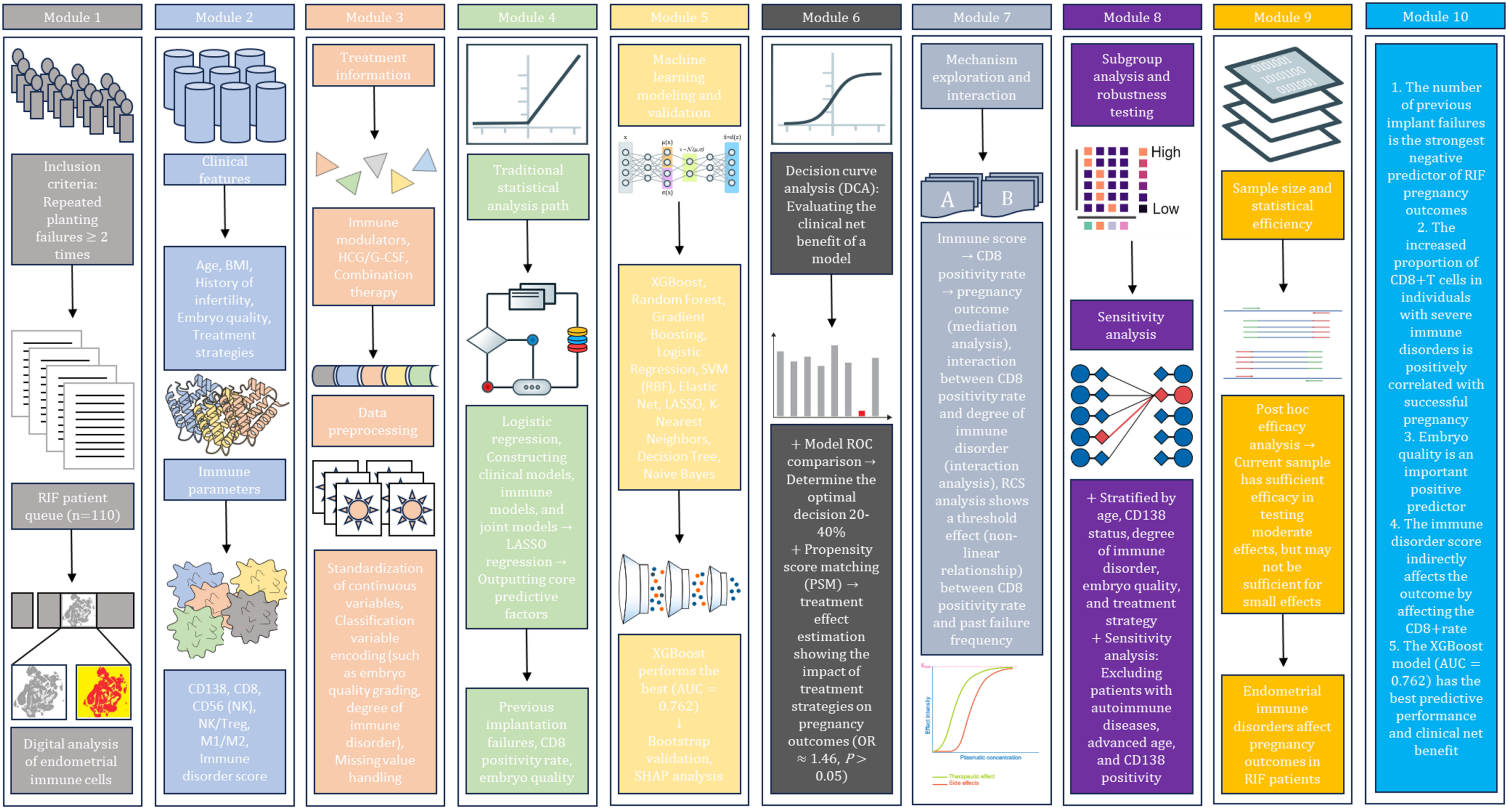
The workflow diagram of this study.

This study adopted an integrated two−phase analytical framework, combining hypothesis guided causal inference with data−driven ML (**Figure 1**). A deeply phenotyped cohort of RIF patients was established, comprising comprehensive demographic, reproductive, endometrial immune, treatment, and embryological data. Predictive modeling and variable selection were performed using both multivariable logistic regression and ML algorithms to identify and validate key predictors of pregnancy success. Validated predictors were carried forward for mechanistic investigation *via* mediation analysis, restricted cubic spline (RCS) modeling for nonlinearity and thresholds, and the subgroup analysis to assess effect heterogeneity. The robustness and clinical utility of the findings were evaluated through sensitivity analyses, bootstrap validation, and decision curve analysis (DCA).

### 2.2 Subjects

A retrospective analysis was performed on 179 women diagnosed with the RIF at the Reproductive Center of Boai Hospital of Zhongshan between January 2023 and December 2025. Following initial consultation and standard etiological screening for genetic, uterine, endocrine, autoimmune disorders. Patients without identifiable causes were recommended for endometrial immune profiling during the mid−luteal phase (24, 25).

From this cohort, the 110 patients who underwent immune analysis of endometrial tissue were included. The remaining 69 patients were excluded due to incomplete immune data (26).

Inclusion criteria were: age < 45 years; and either ≥ 2 pregnancy losses before 20 weeks of gestation, or failure to achieve a clinical pregnancy after ≥ 10 high−grade embryo transfers over 2–6 IVF cycles. Exclusion criteria comprised uterine abnormalities, significant hormonal or metabolic disorders, and clinically diagnosed autoimmune diseases (27, 28).

Among the 110 included patients, 72 (65.5%) met the RIF criteria (failure to achieve clinical pregnancy after ≥ 10 high−grade embryo transfers over 2–6 IVF cycles), 31 (28.2%) met the RPL criteria (≥ 2 pregnancy losses before 20 weeks of gestation), and 7 (6.4%) fulfilled both criteria. The distribution of failure outcomes in the pregnancy failure group (n = 66) was as follows: 32 (48.5%) with no pregnancy, 18 (27.3%) with biochemical pregnancy, 14 (21.2%) with miscarriage, and 2 (3.0%) with ectopic pregnancy or induced abortion. These data are summarized in **Supplementary Table 1**.

### 2.3 Data collection

To establish an integrated clinical-immune predictive framework, multi-dimensional data were systematically collected, including demographics, the reproductive history, a detailed endometrial immune cell atlas, clinical features, and treatment strategies (**Figure 1**; **Table 1**).

**Table 1:** Baseline characteristics of the study population (n = 110).

Characteristic	Overall (n = 110)	Pregnancy Failure (n = 66)	Pregnancy Success (n = 44)	*P*-value
Demographics				
Age (years), Mean ± SD	34.0 ± 4.2	34.3 ± 4.4	33.6 ± 3.9	0.389
Age < 35 years, n (%)	66 (60.0)	39 (59.1)	27 (61.4)	0.805
Age 35–40 years, n (%)	38 (34.5)	23 (34.8)	15 (34.1)	0.938
Age > 40 years, n (%)	6 (5.5)	4 (6.1)	2 (4.5)	0.728
BMI (kg/m²), Mean ± SD	22.4 ± 3.3	22.7 ± 3.5	21.9 ± 3.0	0.203
Infertility history				
Duration of infertility (years), Median (IQR)	3.0 (1.0–6.0)	3.0 (1.0–6.0)	2.0 (1.0–5.0)	0.167
Number of previous miscarriages, Median (IQR)	0.0 (0.0–1.0)	0.0 (0.0–1.0)	0.0 (0.0–1.0)	0.865
Number of previous implantation failures, Median (IQR)	3.0 (2.0–4.0)	4.0 (2.0–5.0)	3.0 (1.0–4.0)	**0.014**
Total number of failures, Median (IQR)	3.0 (2.0–6.0)	4.0 (2.0–7.0)	3.0 (1.0–5.0)	**0.022**
Immune parameters				
CD138 positive, n (%)	4 (3.6)	1 (1.5)	3 (6.8)	0.137
CD138+ rate (%), Median (IQR)	0.0 (0.0–0.0)	0.0 (0.0–0.0)	0.0 (0.0–0.0)	0.712
CD8+ rate (%), Median (IQR)	1.89 (1.46–2.63)	1.83 (1.46–2.55)	2.07 (1.49–2.76)	0.356
CD56+ (NK) rate (%), Median (IQR)	0.058 (0.041–0.078)	0.059 (0.042–0.078)	0.057 (0.040–0.079)	0.650
NK/Treg ratio, Median (IQR)	6.12 (4.10–8.75)	5.88 (3.92–8.65)	6.25 (4.46–9.04)	0.521
M1/M2 macrophage ratio, Median (IQR)	92.5 (62.5–146.0)	94.8 (63.1–153.2)	89.5 (62.5–138.2)	0.742
Immune dysregulation assessment				
Immune disorder score, Mean ± SD	4.73 ± 1.32	4.83 ± 1.33	4.59 ± 1.29	0.336
Degree of immune disorder, n (%)				0.423
Mild (Score 1)	42 (38.2)	23 (34.8)	19 (43.2)	
Moderate (Score 2)	50 (45.5)	32 (48.5)	18 (40.9)	
Severe (Score 3)	15 (13.6)	9 (13.6)	6 (13.6)	
Very severe (Score 4)	3 (2.7)	2 (3.0)	1 (2.3)	
Clinical features				
Autoimmune disease, n (%)	3 (2.7)	1 (1.5)	2 (4.5)	0.336
Embryo quality, n (%)				**0.047**
AA (Grade 1)	24 (21.8)	14 (21.2)	10 (22.7)	
AB (Grade 2)	43 (39.1)	21 (31.8)	22 (50.0)	
BB (Grade 3)	33 (30.0)	24 (36.4)	9 (20.5)	
BC (Grade 4)	9 (8.2)	6 (9.1)	3 (6.8)	
None (Grade 0)	1 (0.9)	1 (1.5)	0 (0.0)	
Treatment strategies				
Treatment category, n (%)				0.416
Immune modulators (Category 1)	12 (10.9)	6 (9.1)	6 (13.6)	
HCG/G-CSF infusion (Category 2)	36 (32.7)	23 (34.8)	13 (29.5)	
Antibiotic therapy (Category 3)	2 (1.8)	1 (1.5)	1 (2.3)	
Combination therapy (Category 4)	52 (47.3)	30 (45.5)	22 (50.0)	
No treatment (Category 5)	8 (7.3)	6 (9.1)	2 (4.5)	
Specific medication use, n (%)				
Cyclosporine	10 (9.1)	6 (9.1)	4 (9.1)	1.000
Prednisone	24 (21.8)	14 (21.2)	10 (22.7)	0.850
HCG infusion	30 (27.3)	17 (25.8)	13 (29.5)	0.667
Hydroxychloroquine	4 (3.6)	2 (3.0)	2 (4.5)	0.687
G-CSF infusion	18 (16.4)	9 (13.6)	9 (20.5)	0.345
Dexamethasone	5 (4.5)	3 (4.5)	2 (4.5)	1.000

Data are presented as mean ± standard deviation (SD), median (interquartile range, IQR), or number (percentage).

Continuous variables were compared using Student’s *t*−test (normal distribution) or Mann–Whitney U test (non−normal distribution); categorical variables were compared using the χ² test or Fisher’s exact test, as appropriate.

Bold values represent P-value < 0.05, which is statistically significant.

Clinical records and laboratory reports were reviewed to extract the following information. Demographic data comprised age and body mass index (BMI). Reproductive history included duration of infertility, number of previous miscarriages and implantation failures, and the total number of failures. Endometrial immune profiles were quantified *via* immunohistochemistry, assessing proportions of immune subsets such as CD138^+^ plasma cells, CD8^+^ T cells, CD56^+^ NK cells, Foxp3^+^ regulatory T cells, CD68^+^ and CD163^+^ macrophages, and CD1a^+^ dendritic cells. Derived indices were calculated. Composite immune dysregulation score was computed from multi-parameter deviations, categorizing patients as mild, moderate, severe, or very severe. Clinical features included autoimmune disease history and embryo quality grade. The treatment data encompassed therapeutic strategies and specific medications (17, 29, 30).

### 2.4 Variable definitions

Categorical and ordinal variables (such as embryo quality grade, immune disorder degree, treatment category) are defined. The cumulative burden of reproductive failure was assessed using the number of previous implantation failures and the total number of failures.

To quantify the overall degree of endometrial immune dysregulation, we constructed a composite immune disorder score based on eight key immune cell subsets quantified by IHC: CD8^+^ T cells, CD56^+^ NK cells, Foxp3^+^ Tregs, CD68^+^ and CD163^+^ M2 macrophages, CD1a^+^ immature dendritic cells, CD138^+^ plasma cells, and the NK/Treg ratio. These parameters were selected because they represent major innate and adaptive immune components implicated in endometrial receptivity and implantation (31–33).

A comprehensive immune dysregulation score was calculated as: Σ| (X_i_ − μ_i_)/σ_i_ |, where X_i_ is the patient’s value for each immune parameter, μ_i_ is the population median, and σ_i_ is the population standard deviation. The immune disorder score was then calculated as the sum of these eight standardized deviations. A higher score indicates a more pronounced overall immune deviation. Based on pre-specified cut-offs, the score was categorized ordinally as: I (0–3.5, normal/mild), II (3.5–5.5, moderate), III (5.5–7, severe), or IV (> 7, very severe).

The primary outcome was pregnancy success, defined as ultrasound-confirmed clinical pregnancy after embryo transfer. Pregnancy failure included all other outcomes, such as non-pregnancy, biochemical pregnancy, miscarriage, ectopic pregnancy, or induced abortion.

### 2.5 Endometrial biopsy

All endometrial biopsies were obtained prospectively at the time of study enrollment, after the diagnosis of RIF/RPL and before any immune−based therapy for the subsequent IVF cycle.

The sampling was performed during the mid−luteal phase (LH + 7–9 days) of a natural or hormone−replacement cycle, prior to the embryo transfer that defined the primary outcome. Immune parameters represent baseline predictors, unaffected by subsequent treatments. The timing of immune profiling relative to key clinical events is summarized in **Supplementary Table 2**.

Endometrial samples were collected in the midluteal phase (LH-day 7–9) (34). Endometrial tissue was obtained using a Gynetics endometrial curette, and all samples were processed within 2h of collection. Following collection, specimens were fixed in 10% neutral-buffered formalin for 4–6h at room temperature before paraffin embedding.

### 2.6 Immunohistochemistry staining and image collection and analysis

**Figure 2:**
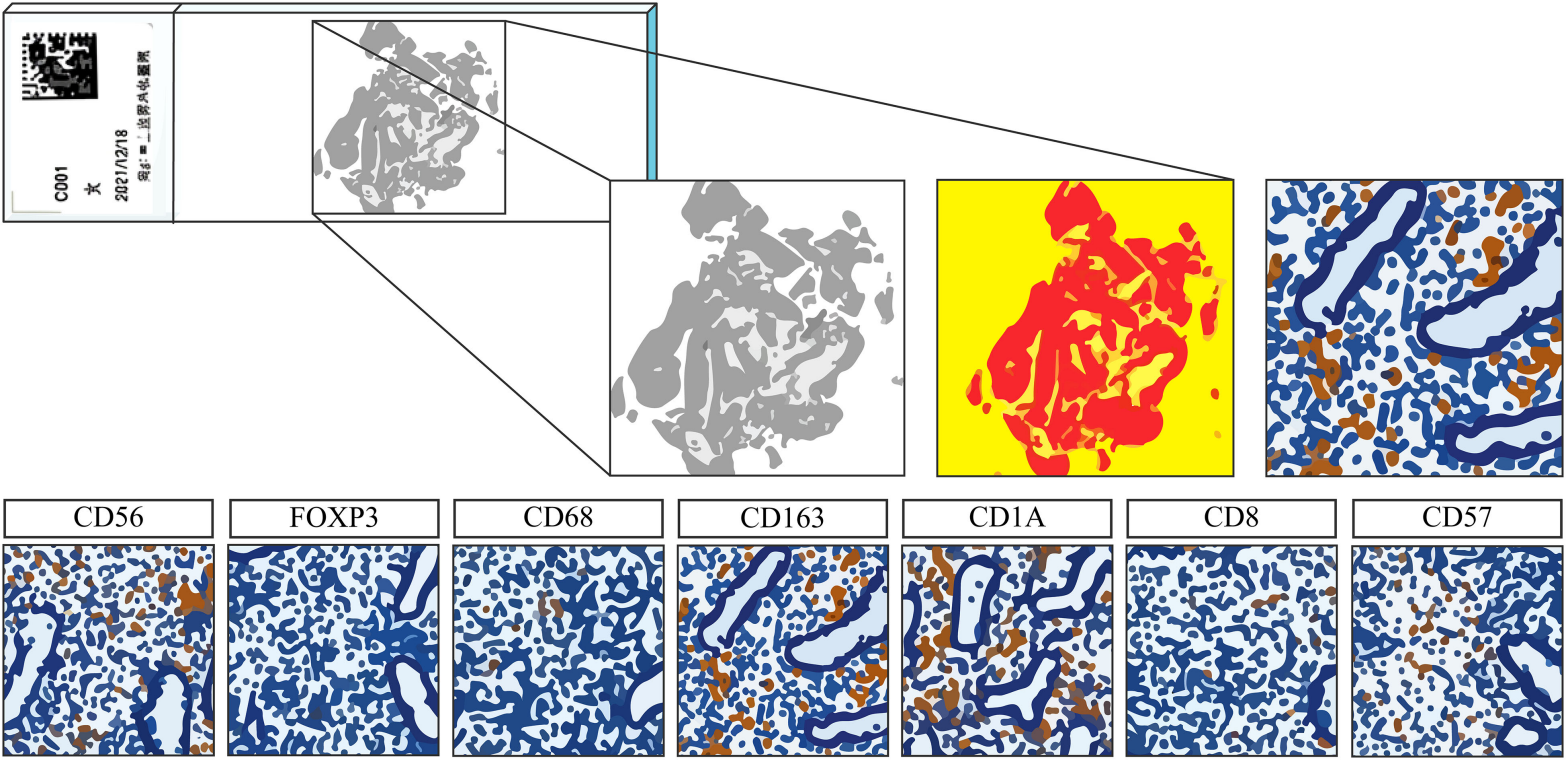
Schematic diagram of digital analysis of endometrial immune cells.

Paraffin-embedded endometrial tissues were sectioned at 4μm. One randomly selected section per sample underwent hematoxylin and eosin (HE) staining for histological dating according to a standardized 28−day cycle. The immunohistochemistry (IHC) was performed automatically on a Leica Bond III immunostainer. Immune cell quantification was conducted using a PerkinElmer analysis system. Whole slides were scanned at low magnification, and 30 random high−power fields (HPFs) per section were imaged. Under pathological supervision, immune cell populations were characterized and quantified *via* the cell segmentation and phenotyping using Inform Cell Analysis software (PerkinElmer). Proportion of each immune subset was calculated as a percentage of all endometrial cells across the 30 HPFs (**Figure 2**).

The endometrial tissues were stained with the antibodies: Anti-CD138 (1:100), Anti-CD56 (1:200) (Gene Tech, GT212629, GT200529), Anti-CD68 (1:100), Anti-CD163 (1:1200), Anti-CD1a (1:30), Anti-CD8 (1:150) (Novocastra, NCL-CD68-KP1, NCL-L-CD163, NCL-CDla-235, NCL-L-CD8-4B11), Anti-Foxp3 (1:100) (eBioscience, 14-4777), Anti-CD83 (1:150) (Sigma-Aldrich, HPA041454). All immune data presented in this manuscript (including CD8^+^ T cells, CD56^+^ NK cells, Foxp3^+^ Tregs, and other subsets) are derived exclusively from endometrial tissue IHC.

### 2.7 Diagnosis of chronic endometritis

For the assessment of chronic endometritis (CE), CD138^+^ plasma cells in the endometrial stroma were quantified by light microscopy across 30 randomly selected high−power fields (HPFs; ×400).

Diagnosis adhered to a criterion from a prior prospective study, wherein patients with ≥5 CD138^+^ cells in any single HPF showed a significantly lower clinical pregnancy rate (26). Cases with ≥ 5 CD138^+^ cells in at least one HPF were classified as CE (35, 36); those with fewer than five cells in every HPF were considered non−CE.

### 2.8 Statistical modeling framework

The association between individual variables and pregnancy success was first assessed using univariate logistic regression to calculate unadjusted odds ratios (Ors) with the 95% confidence intervals (Cis). To identify a parsimonious set of predictors and mitigate overfitting, variable selection was performed *via* Least Absolute Shrinkage and Selection Operator (LASSO) regression with 10−fold cross−validation, selecting the penalty parameter (λ) that minimized the mean cross−validated error (37). Guided by clinical reasoning and the LASSO−selected variables, three multivariable logistic regression models were then constructed: a clinical factor model, an immune factor model, and a combined model. Model discrimination was evaluated using the area under the receiver operating characteristic curve (AUC), and model fit was assessed with the Akaike Information Criterion (AIC) and the Hosmer–Lemeshow goodness−of−fit test (38, 39). Results are reported as adjusted odds ratios (aORs) with 95% Cis.

### 2.9 ML predictive modeling and interpretability

Ten supervised ML classifiers were implemented: Gradient Boosting Machine, XGBoost, Random Forest, Logistic Regression, SVM (RBF), Elastic Net, Lasso, K−Nearest Neighbors, Decision Tree, and Naïve Bayes. Hyperparameter tuning was performed via grid search within a nested 5−fold cross−validation framework, optimized for AUC. Final models were trained using the optimal hyperparameters and evaluated using AUC, accuracy, sensitivity, specificity, F1 score, and Brier score (**Supplementary Table 3**) (40, 41).

To interpret the best−performing model (XGBoost) (42, 43), Shapley Additive exPlanations (SHAP) analysis was employed (44, 45). This method quantifies the marginal contribution of each feature to individual predictions, providing global and local interpretability. Global feature importance was ranked by mean absolute SHAP value. The SHAP dependence and interaction values were further used to visualize directional effects and identify significant feature interactions (46).

### 2.10 Mediation analysis

Causal mediation analysis tested whether the effect of overall immune dysregulation on pregnancy outcome was mediated by specific immune populations, particularly CD8^+^ T−cell proportions (47, 48). Using regression−based bootstrap approach, total effect was decomposed into a direct (immune score → outcome) and an indirect effect via the mediator (immune score → CD8^+^ → outcome) (49). The proportion mediated was calculated as the indirect/total effect ratio. This framework was extended to parallel multiple mediation to assess contributions of other immune parameters. Subgroup−specific analyses were performed within age and immune−disorder strata to evaluate pathway robustness.

### 2.11 Interaction and subgroup analyses

To examine effect heterogeneity, interaction terms were incorporated into the final multivariable model, with significance assessed *via* likelihood ratio tests. Where interactions were significant, models were stratified to the estimate stratum-specific aORs (*P* < 0.05). Prespecified subgroup analyses were conducted by stratifying the cohort by age (< 35 vs. ≥ 35 years), CD138 status, immune disorder severity (mild/moderate vs. severe/very severe), embryo quality (good [AA/AB] vs. poor [BB/BC/None]), and primary treatment strategy (immune-based vs. other).

### 2.12 Nonlinearity and threshold effects

Nonlinear relationships between continuous predictors and the log-odds of success were examined using RCS with three knots. Overall significance and nonlinearity of each smoothed relationship were tested. Where a nonlinear trend or inflection point was evident, piecewise effect estimates were derived for interpretable ranges, and potential thresholds were formally tested by comparing linear and segmented linear models (50). All RCS analyses were adjusted for other covariates in the final model.

### 2.13 Model validation, calibration, and decision curve analysis

Internal validation was performed using 1000 bootstrap resamples. For each primary model, optimism (the performance difference between bootstrap and out−of−bag samples) was estimated and subtracted from the apparent performance to obtain optimism−corrected estimates of the AUC, calibration slope, and Brier score.

Calibration was assessed *via* calibration−in−the−large, calibration slope, and Harrell’s E90 statistic across risk deciles (51, 52). The clinical utility of each model was evaluated using DCA. DCA quantifies the net clinical benefit of using a prediction model to guide treatment decisions across a range of threshold probabilities (53). In this analysis, the clinical action of interest was the decision to administer immune-based therapy (as defined in Section 2.4). The prediction target was the probability of pregnancy success. For a given threshold probability *Pt*, it was assumed that a clinician would choose to intervene if the model-predicted probability of success was below *Pt*​, reflecting the rationale that patients with a low predicted chance of success might benefit from additional intervention.

Conversely, if the predicted probability was above the P_t_, no intervention would be offered. Under this assumption, a true positive was defined as a patient with predicted success probability < P_t_​ who actually experienced pregnancy failure, and a false positive as a patient with predicted success probability < P_t_ who actually achieved pregnancy success. The net benefit was calculated as:


$$Net\;Benefit\;=\;True\;Positives/N–\;False\;Positives/N\times \;P_{t}/\left(1\;–\;P_{t}\right)$$

The net benefit of each model−based strategy was compared to the treat all and treat none strategies, with results presented as net benefit and standardized net benefit curves across thresholds from 10% to 80%.

### 2.14 Sensitivity analysis

To assess the robustness of the primary findings, three pre−specified sensitivity analyses were conducted. First, patients with diagnosed autoimmune diseases (n = 3) were excluded to evaluate whether such conditions influenced the observed associations. Second, patients older than 40 years (n = 6) were excluded to assess the potential impact of advanced maternal age. Third, the analysis was restricted to CD138−negative patients (n = 106) to determine whether the presence of chronic endometritis (defined by CD138 positivity) affected the results. In each sensitivity analysis, the final multivariable logistic regression model was refitted, which included the five LASSO−selected predictors (number of previous implantation failures, total number of failures, embryo quality grade, CD8^+^ T−cell proportion, and BMI).

### 2.15 Propensity score analysis

To assess the potential causal effect of immune−based therapy, propensity score methods (PSM) were employed (54). Propensity scores were estimated using multivariable logistic regression with baseline covariates including age, BMI, number of previous implantation failures, total number of failures, embryo quality grade, CD8^+^ T−cell proportion, and immune disorder score (55). The distribution of propensity scores was examined for overlap between treatment groups to ensure common support; patients with propensity scores outside the overlapping region were excluded from matched analyses.

1:1 nearest−neighbor matching without replacement, with a caliper width of 0.2 times the standard deviation of the logit propensity score. Standardized mean differences (SMD) were calculated to assess covariate balance, with SMD < 0.1 considered acceptable.

Caliper matching with a caliper of 0.2 SD, also without replacement.

Inverse probability of treatment weighting (IPTW) using stabilized weights to reduce variability. Weights were truncated at the 1st and 99th percentiles to limit the influence of extreme weights. Balance after weighting was assessed using weighted standardized mean differences.

The average treatment effect on the treated (ATT) was estimated as the risk difference in the matched sample and as weighted risk differences in the IPTW analysis. E−values were calculated to assess the potential impact of unmeasured confounding (56, 57).

### 2.16 Consistency and consensus analysis of multi-model predictions

The agreement among the five core predictive models in classifying outcomes was assessed using Fleiss’ Kappa. Consistency of predicted risk scores was quantified with the Intraclass Correlation Coefficient (ICC) for absolute agreement (58, 59). Detailed distribution of model agreement is presented in Table, with cases stratified into five categories based on the level of consensus: unanimous success (5/5 models predicting success), majority success (≥ 3/5 models predicting success), divided prediction (2–3 models predicting success and 2–3 predicting failure), majority failure (≥ 3/5 models predicting failure), and unanimous failure (5/5 models predicting failure).

For comparative analysis of clinical and immune characteristics, cases were dichotomized into consistent predictions (defined as ≥ 3 of 5 models agreeing on the outcome, including unanimous and majority categories) and discordant predictions (defined as < 3 models agreeing, corresponding to the divided prediction category). This dichotomization allows for the identification of factors associated with predictive uncertainty.

### 2.17 Sample size and *post-hoc* power considerations

A *post-hoc* power analysis was conducted based on the observed effect sizes of the primary predictors in the final multivariable model to quantify the statistical power of the current study sample (n = 110). Furthermore, to inform the design of future validation or mechanistic studies, sample size estimations were performed. These calculations determined the number of participants required to achieve 80% and 90% statistical power for detecting effect sizes ranging from large to small, assuming a two-sided alpha level of 0.05 (60).

### 2.18 Statistic analysis

All analyses were conducted in R (versions 4.1.3 and 4.3.2). Categorical variables are presented as frequencies (percentages) and compared using the χ² test or Fisher’s exact test. Between−group comparisons were made with Student’s t−test (normally distributed data) or the Mann–Whitney U test (non−normally distributed data), with normality assessed *via* the Shapiro–Wilk test.

## 3 Results

### 3.1 Cohort characteristics and univariate associations with pregnancy success

Immune profiling was performed at baseline (after diagnosis but before any treatment for the index cycle), ensuring that immune parameters reflect pre−intervention status (**Supplementary Table 2**).

The study included 110 women with RIF, of whom 44 (40.0%) achieved pregnancy success (**Table 1**). Mean age of participants was 34.0 ± 4.2 years, and the mean BMI was 22.4 ± 3.3 kg/m², with no significant differences between the outcome groups. Reproductive history revealed a higher median number of previous implantation failures and a higher total number of failures (implantation failures plus miscarriages) in the pregnancy failure group compared to the success group (4.0 [IQR 2.0–5.0] vs. 3.0 [1.0–4.0], *P* = 0.014; and 4.0 [2.0–7.0] vs. 3.0 [1.0–5.0], *P* = 0.022, respectively). Embryo quality distribution differed significantly between groups (*P* = 0.047), with a higher proportion of good-quality embryos (AB) in the success group. The prevalence of CE (CD138 positivity) was low overall (3.6%) and did not differ significantly between the groups.

In univariate logistic regression analysis (**Supplementary Table 4**), each additional previous implantation failure was associated with a 21% reduction in the odds of pregnancy success (OR 0.79, 95% CI 0.66–0.94, *P* = 0.008). Similarly, each additional unit increase in the total number of failures reduced the odds by 8% (OR 0.92, 95% CI 0.85–0.99, *P* = 0.040).

Embryo quality, when analyzed as an ordinal variable, did not show a statistically significant association with pregnancy success in univariate analysis (**Supplementary Table 4**). However, after adjusting for confounders in the multivariable model, each one−grade improvement in embryo quality was independently associated with a 62% increase in the odds of success (aOR = 1.62, 95% CI 1.04–2.53, *P* = 0.033) (**Table 2**).

**Table 2:** Final multivariable model based on LASSO selection.

Variable	aOR (95% CI)	*P*-value
Previous implantation failures (per failure)	0.74 (0.60-0.91)	**0.004**
Total number of failures (per failure)	0.94 (0.86-1.02)	0.135
Embryo quality (per grade improvement)	1.62 (1.04-2.53)	**0.033**
CD8 rate (per 1% increase)	1.25 (1.03-1.52)	**0.025**
BMI (per kg/m² increase)	0.94 (0.85-1.04)	0.225

Bold values represent P-value < 0.05, which is statistically significant.

**Figure 3:**
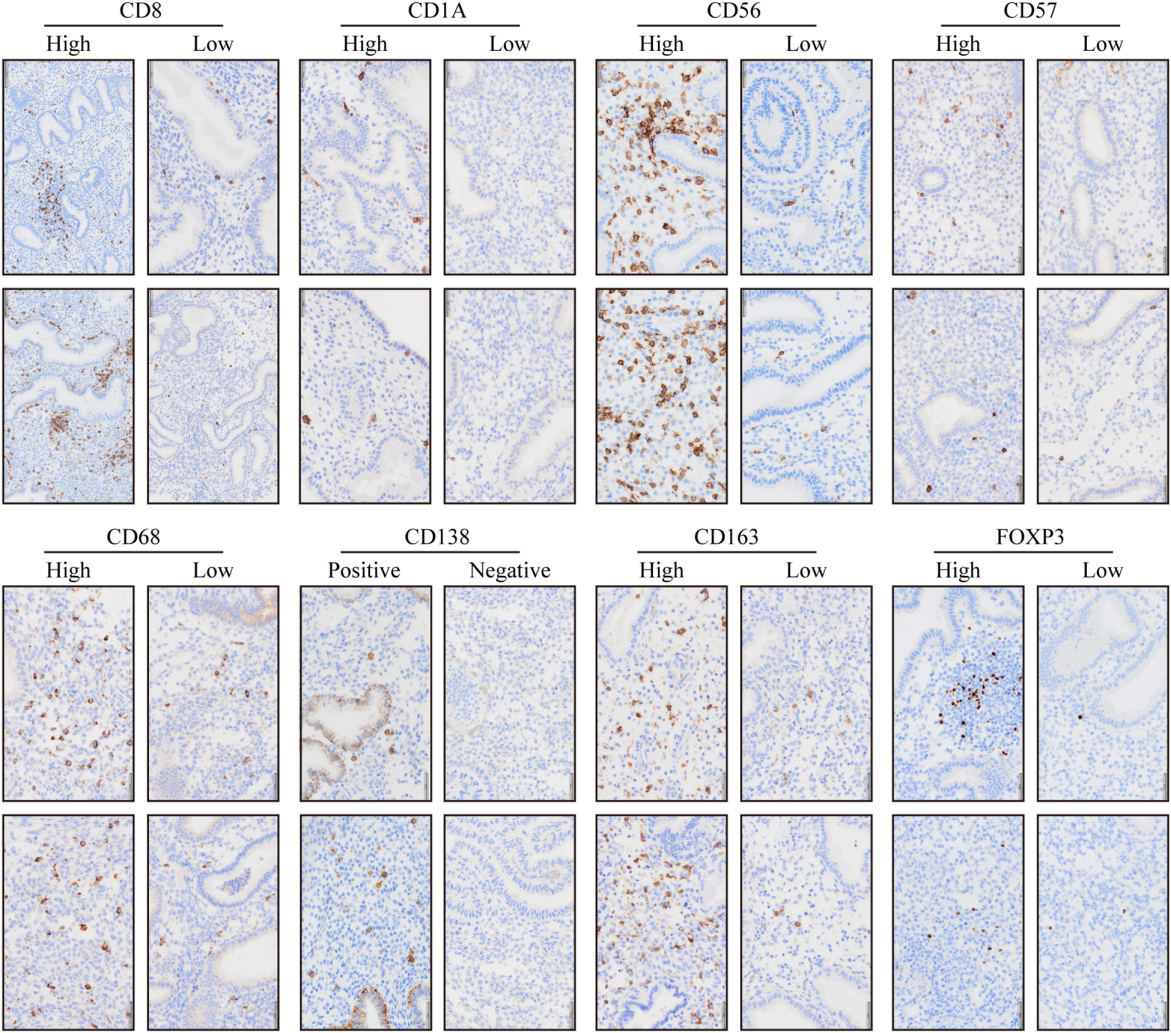
Endometrial immune cells in patients with recurrent reproductive failure with and without CE. Immunostaining was performed on endometrial biopsies from RIF patients (with or without CE) to evaluate uterine CD56+ natural killer cells, CD68+ macrophages, CD163+ M2 macrophages, CD1a+ immature dendritic cells, CD83+ mature dendritic cells, CD8+ T cells, and Foxp3+ Tregs.

IHC staining of endometrial biopsies illustrated in−situ distribution of key innate immune cells evaluated in this cohort, including CD8^+^ T, CD56^+^ NK cells, CD68^+^ macrophages, CD163^+^ M2 macrophages, CD1a^+^ immature dendritic, CD138^+^ plasma cells, and Foxp3^+^ Tregs (**Figure 3**). Representative images depicted high versus low expression patterns for major markers, alongside positive versus negative staining for CD138, aligning with the quantitative profiles summarized in **Table 1**.

The pregnancy failure group comprised 32 (48.5%) with no pregnancy, 18 (27.3%) with biochemical pregnancy, 14 (21.2%) with miscarriage, and 2 (3.0%) with ectopic pregnancy or induced abortion (**Supplementary Table 1**). Among the entire cohort, 72 patients (65.5%) met the strict RIF definition, 31 (28.2%) met the RPL definition, and 7 (6.4%) met both.

### 3.2 Predictor selection and multivariable logistic regression models

LASSO selected five variables: number of previous implantation failures, total number of failures, embryo quality grade, CD8^+^ T−cell proportion, and BMI (**Supplementary Table 5**). These variables were carried forward for multivariable modeling.

The clinical factors model, containing age, BMI, previous implantation failures, and embryo quality, demonstrated moderate discrimination (AUC 0.68, 95% CI 0.58–0.78). The immune factors model, containing CD138 status, CD8^+^ T−cell proportion, NK/Treg ratio, and immune disorder score, showed lower discriminative performance (AUC 0.62, 95% CI 0.51–0.73). The combined model, integrating the LASSO−selected predictors, achieved highest performance among regression models (AUC 0.71, 95% CI 0.61–0.81) and exhibited adequate calibration (Hosmer−Lemeshow *P* = 0.385) (**Supplementary Tables 6**, **7**).

Each additional previous implantation failure was independently associated with a 26% reduction in the adjusted odds of pregnancy success (aOR 0.74, 95% CI 0.60–0.91, *P* = 0.004) (**Table 2**). In contrast, each 1% increase in endometrial CD8^+^ T−cell proportion was associated with a 25% increase in the adjusted odds of success (aOR 1.25, 95% CI 1.03–1.52, *P* = 0.025). Each incremental improvement in embryo quality grade nearly doubled the odds of success (aOR 1.62 per grade, 95% CI 1.04–2.53, *P* = 0.033) (**Supplementary Table 8**).

### 3.3 ML model performance and comparison

After hyperparameter optimization *via* nested cross−validation, model performance was evaluated across a comprehensive set of metrics (**Table 3**; **Supplementary Table 9**).

**Table 3:** Comparison of 10 machine learning methods for predicting pregnancy success.

Method	AUC (95% CI)	Accuracy	Sensitivity	Specificity	F1 Score	Brier score
XGBoost	0.762 (0.734-0.790)	72.7%	74.1%	71.8%	0.713	0.189
Random Forest	0.751 (0.721-0.781)	71.8%	72.7%	71.2%	0.701	0.195
Gradient Boosting	0.745 (0.715-0.775)	70.9%	71.8%	70.3%	0.694	0.201
Logistic Regression	0.738 (0.707-0.769)	69.1%	70.5%	68.2%	0.682	0.208
SVM (RBF)	0.731 (0.699-0.763)	68.2%	69.1%	67.6%	0.673	0.212
Elastic Net	0.728 (0.696-0.760)	67.3%	68.2%	66.7%	0.668	0.215
LASSO	0.725 (0.692-0.758)	66.4%	67.3%	65.9%	0.662	0.218
K-Nearest Neighbors	0.703 (0.668-0.738)	65.5%	65.9%	65.2%	0.647	0.228
Decision Tree	0.691 (0.655-0.727)	64.5%	65.5%	63.6%	0.635	0.235
Naive Bayes	0.658 (0.620-0.696)	62.7%	63.6%	62.1%	0.614	0.251

The XGBoost algorithm achieved the highest discriminative performance, with an AUC of 0.762 (95% CI 0.734–0.790), an accuracy of 72.7%, and the lowest Brier score (0.189). Other tree−based ensemble methods, including Random Forest (AUC 0.751) and Gradient Boosting Machine (AUC 0.745), also demonstrated strong performance. Traditional regression−based models (Logistic Regression, LASSO, Elastic Net) and SVM showed intermediate performance, while simpler models (K−Nearest Neighbors, Decision Tree, Naïve Bayes) exhibited more modest predictive capability.

Feature importance analysis within the best−performing XGBoost model corroborated the central role of clinical history and specific immune parameters (**Supplementary Table 10**). The number of previous implantation failures was the most influential predictor (importance score 38.4%), followed by CD8^+^ T−cell proportion (25.6%) and embryo quality (19.2%). The total number of failures and BMI contributed smaller but non−negligible shares to the model’s predictions.

SHAP confirmed the negative directional effect of previous failures and the positive effect of CD8^+^ T−cell proportion on predicted probability (**Supplementary Table 11**). Furthermore, SHAP dependence and interaction analysis revealed that a higher CD8^+^ T−cell proportion could partially mitigate the negative impact of multiple previous failures, and that its protective effect was more pronounced within the normal BMI range (**Supplementary Tables 12**, **13**).

### 3.4 Mediation role of CD8^+^ T cells between immune dysregulation and outcome

Causal mediation analysis revealed a significant total effect of the immune disorder score on reducing the probability of pregnancy success (coefficient −0.184, *P* = 0.039). This total effect was decomposed into a direct effect (immune score → outcome) and an indirect effect mediated through CD8^+^ T−cell proportion (**Table 4**).

**Table 4:** Mediation analysis of CD8 rate on the effect of immune disorder score.

Path/Effect	Coefficient	Standard Error	95% CI	*P*-value	Proportion Mediated
Total Effect	-0.184	0.089	(-0.358, -0.010)	0.039	100.0%
Direct Effect	-0.142	0.091	(-0.320, 0.036)	0.119	77.2%
Indirect Effect (through CD8_rate)	-0.042	0.024	(-0.089, 0.005)	0.079	22.8%
Path a (Immune score → CD8 rate)	-0.156	0.082	(-0.317, 0.005)	0.058	/
Path b (CD8 rate → Outcome)	0.269	0.120	(0.034, 0.504)	0.025	/

The indirect effect through CD8^+^ T cells was −0.042 (95% CI −0.089 to 0.005), accounting for 22.8% of the total effect. The proportion mediated indicated that a substantial share of the impact of systemic immune dysregulation operates by influencing the endometrial CD8^+^ T−cell compartment. Path analysis confirmed a negative association between the immune disorder score and CD8^+^ T−cell proportion (a: coefficient −0.156, *P* = 0.058), and a positive association between CD8^+^ T−cell proportion and pregnancy success (b: coefficient 0.269, *P* = 0.025).

Expanding to a parallel multiple mediation model (**Supplementary Table 14**) showed that other immune parameters, including the NK/Treg ratio and M1/M2 macrophage ratio, contributed minor additional indirect paths. The total indirect effect through all considered immune mediators was −0.078, explaining 42.4% of the total effect of immune dysregulation.

Subgroup−specific mediation analyses demonstrated that the mediated pathway *via* CD8^+^ T cells was not uniform across clinical phenotypes (**Supplementary Table 15**). It was most pronounced in patients with severe/very severe immune disorder, where it accounted for 38.9% of the total effect (*P* = 0.038). The mediated proportion was smaller and non−significant in patients with mild/moderate disorder, and consistent but non−significant across age and CD138 subgroups.

### 3.5 Interaction effects and subgroup heterogeneity

A significant interaction was identified between endometrial CD8^+^ T−cell proportion and the degree of immune disorder (*P* = 0.034) (**Table 5**). Stratified analysis revealed that the positive association between CD8^+^ T−cell proportion and pregnancy success was markedly stronger in patients with severe or very severe immune disorder (aOR 1.52 per 1% increase, 95% CI 1.13–2.04) compared to those with mild or moderate disorder (aOR 1.15, 95% CI 0.94–1.41).

**Table 5:** Interaction analysis for key predictors.

Interaction term	aOR (95% CI)	*P*-value for interaction
CD8 rate × Previous failures	1.01 (0.98-1.04)	0.482
CD8 rate × Age group		0.218
Age < 35	1.20 (0.92-1.56)	/
Age ≥ 35	1.33 (1.05-1.69)	/
CD8 rate × Immune disorder		0.034
Mild/moderate disorder	1.15 (0.94-1.41)	/
Severe/extremely severe disorder	1.52 (1.13-2.04)	/
Previous failures × Embryo quality	0.99 (0.86-1.14)	0.887

The protective association of CD8^+^ T−cell proportion was consistently positive across most subgroups but reached statistical significance primarily in patients receiving immune−based therapy (aOR 1.32, 95% CI 1.05–1.66) and in those with severe immune disorder, as noted above (**Supplementary Tables 16**-**20**). Detrimental effect of previous implantation failures remained robust and significant across nearly all subgroups, including when stratified by age, CD138 status, embryo quality, and treatment strategy. The benefit of the better embryo quality was most pronounced in younger patients (age < 35 years: aOR 1.85, 95% CI 1.08–3.17) and in those receiving immune−based therapy (aOR 1.75, 95% CI 1.05–2.92).

**Figure 4:**
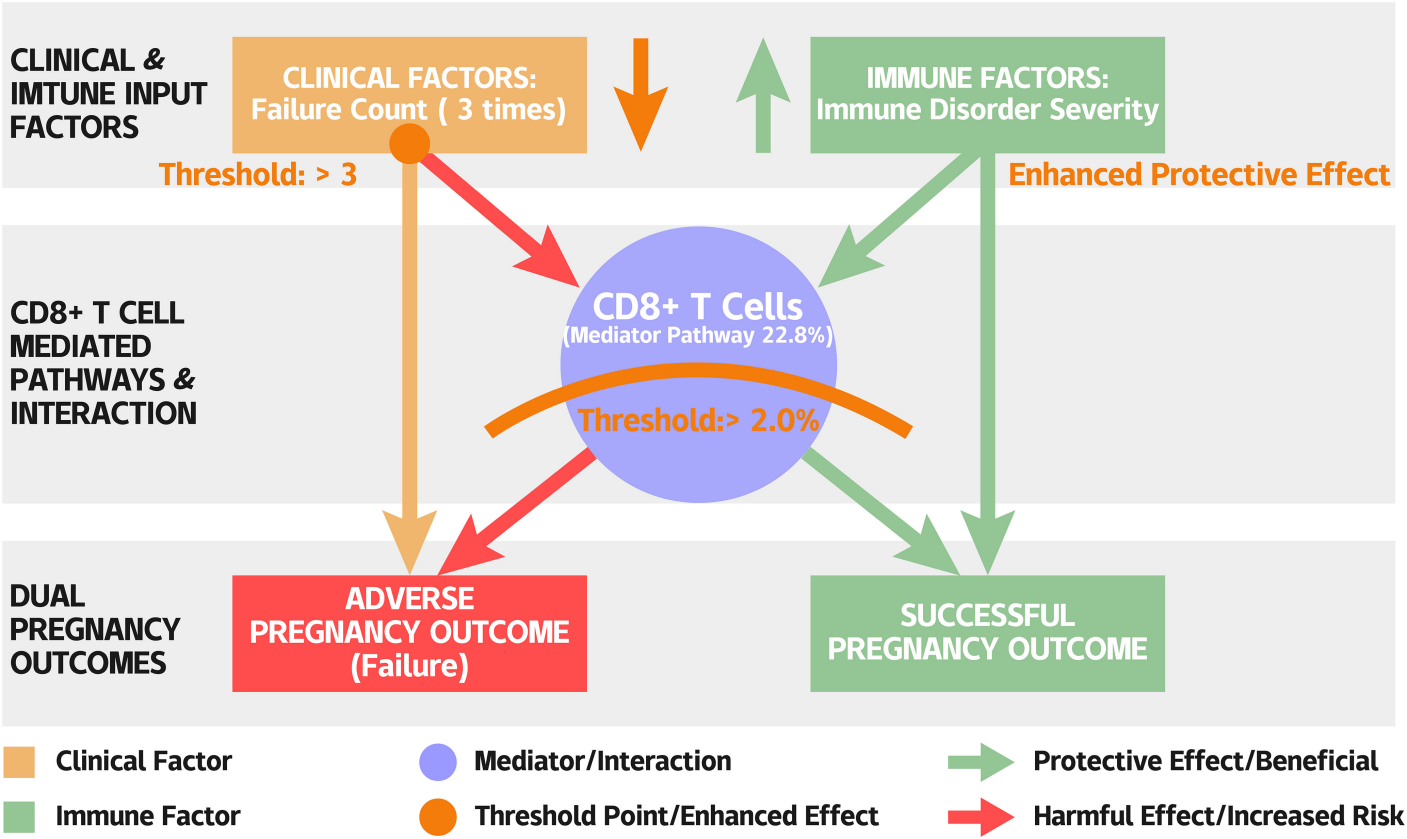
A schematic summary of the integrated clinical-immune mechanism in RIF.

Model performance varied across subgroups, being highest in patients with severe immune disorder (0.865) and in those who were CD138 positive (0.952), though the latter subgroup was small (n = 4). The CD8^+^ T−cell proportion severs as a central, modifiable node whose prognostic impact is contingent upon the severity of the background immune milieu, while the burden of the previous failures exerts a consistently negative effect across diverse patient phenotypes (**Figure 4**).

### 3.6 Nonlinear relationships and threshold effects of continuous predictors

The overall associations for the number of previous implantation failures and CD8^+^ T−cell proportion were statistically significant (*P* = 0.008 and *P* = 0.025), while those for age, BMI, immune disorder score, and other parameters were not (**Table 6**).

**Table 6:** RCS analysis for nonlinear relationships of continuous variables (n = 110).

Variable	Overall P-value	Nonlinear *P*-value	Deviance explained	Optimal knot position
Age (years)	0.205	0.387	3.20%	30, 34, 38
BMI (kg/m²)	0.166	0.231	4.80%	19, 22, 26
Previous failures (failures)	0.008	0.125	12.30%	1, 3, 5
CD8 rate (%)	0.025	0.187	8.70%	1.2, 1.9, 2.6
Immune score (points)	0.255	0.412	5.10%	3.5, 4.7, 5.9
NK Treg rate	0.685	0.543	2.30%	4.0, 6.5, 9.0
Duration infertility (years)	0.157	0.298	4.20%	1, 3, 6

Notably, the relationship between CD8^+^ T−cell proportion and pregnancy success exhibited a nonlinear, threshold−dependent pattern. RCS was relatively flat below approximately 2.0%, after which each incremental increase was associated with a markedly steeper rise in the odds of success (**Supplementary Table 21**). Formal testing for a threshold effect confirmed a significant change in slope at 2.0% (*P* = 0.024), with the odds ratio for success being 40.7% higher above this cut−point (OR 1.52, 95% CI 1.18–1.96) compared to below it (OR 1.08, 95% CI 0.86–1.36) (**Supplementary Table 22**).

Similarly, negative association with the number of previous implantation failures followed a nonlinear, accelerating pattern. The detrimental effect per failure was significantly more pronounced after exceeding three previous failures (*P* = 0.011). Patients with more than five failures had an odds of success only 42% (OR 0.42, 95% CI 0.26–0.68) of those with fewer failures.

### 3.7 Robustness of predictions: model validation, calibration and clinical utility

The XGBoost model maintained the highest corrected discriminative ability (0.727, 95% CI 0.660–0.794), followed by the Combined regression model (0.710, 0.642–0.778) (**Supplementary Table 23**). Calibration performance, assessed across metrics including the calibration slope and Harrell’s E90 statistic, was satisfactory for all models, with combined and final LASSO models showing the best calibration (calibration slopes 0.91 and 0.92, respectively) (**Supplementary Table 24**).

Moreover, the XGBoost model provided the highest net benefit within the clinically most relevant threshold range of 20–40%, outperforming both the default strategies of treat all and treat none as well as all other regression−based models (**Table 7**; **Supplementary Table 25**). This superior net benefit was consistent across the evaluated threshold spectrum (10–80%) and was reflected in both the absolute and standardized net benefit metrics.

**Table 7:** Clinical DCA results (n = 110).

Threshold probability	Net benefit	Standardized net benefit	Treat all strategy	Treat none strategy
10%	0.28	0.18	0.10	0.00
20%	0.32	0.20	0.12	0.00
30%	0.28	0.17	0.14	0.00
40%	0.21	0.13	0.16	0.00
50%	0.15	0.09	0.18	0.00
60%	0.10	0.06	0.20	0.00
70%	0.06	0.04	0.22	0.00
80%	0.03	0.02	0.24	0.00

### 3.8 Sensitivity analyses

After excluding patients with autoimmune diseases (n = 3), the adjusted odds ratios for previous failures (aOR 0.73, 95% CI 0.59–0.90), CD8^+^ T−cell proportion (aOR 1.27, 95% CI 1.04–1.55), and embryo quality (aOR 1.61, 95% CI 1.03–2.52) remained similar to the primary analysis, with a model AUC of 0.741. After excluding patients older than 40 years (n = 6), consistent estimates were also observed (previous failures: aOR 0.73, 95% CI 0.59–0.90; CD8^+^ T−cell proportion: aOR 1.24, 95% CI 1.02–1.52; embryo quality: aOR 1.65, 95% CI 1.05–2.58; AUC 0.736). When the analysis was restricted to CD138−negative patients (n = 106), the effect sizes for previous failures (aOR 0.75, 95% CI 0.61–0.93) and embryo quality (aOR 1.58, 95% CI 1.00–2.48) remained significant, while the association for CD8^+^ T−cell proportion was slightly attenuated and no longer statistically significant (aOR 1.21, 95% CI 0.99–1.48, *P* = 0.064). The model AUC was 0.726 (**Supplementary Table 8**).

### 3.9 Assessment of treatment effect using propensity score matching

Propensity score distributions showed substantial overlap between the treatment and control groups, with all patients falling within the common support region; therefore, no patients were excluded due to non−overlap.

After 1:1 nearest−neighbor matching without replacement (caliper = 0.2 SD), 40 treated patients were successfully matched to 40 control patients (**Supplementary Table 27**). Covariate balance was excellent, with all standardized mean differences ≤ 0.05 after matching. The estimated ATT was a 12.5 percentage−point increase in the probability of pregnancy success (risk difference +12.5%, 95% CI −7.0% to +32.0%, *P* = 0.219) (**Supplementary Table 28**).

Results from caliper matching (risk difference +10.1%, 95% CI −6.8% to +27.0%) and stabilized IPTW with weight truncation (risk difference +9.0%, 95% CI −7.8% to +25.8%) were consistent in direction but similarly imprecise, with all confidence intervals crossing zero.

E−value analysis indicated that an unmeasured confounder would need to be associated with both treatment assignment and outcome by an odds ratio of at least 1.98 to fully explain away the observed point estimate, suggesting moderate robustness to hidden bias (**Supplementary Table 26**).

### 3.10 Consistency across prediction models and analysis of discordant cases

Multi−rater agreement among the five core models was moderate (Fleiss’ Kappa = 0.42, 95% CI 0.35–0.49), while the consistency of predicted risk scores across models was good (ICC = 0.65, 95% CI 0.57–0.72) (**Supplementary Table 30**). The detailed distribution of model agreement is shown in **Supplementary Table 29**. Predictions were unanimous (5/5 models agreeing) in the 23 cases (20.9%), including 18 cases of unanimous success and 5 cases of unanimous failure. Majority consensus (≥ 3/5 models agreeing) was observed in an additional 51 cases (46.4%), comprising 32 cases of majority success and the 19 cases of majority failure. Divided predictions (2–3 models predicting success and 2–3 predicting failure) occurred in 36 cases (32.7%).

For comparative analysis, cases were dichotomized into consistent predictions (≥ 3/5 models agreeing, n = 74) and discordant predictions (< 3 models agreeing, n = 36). Cases with discordant predictions exhibited distinct clinical−immune profiles compared to those with consistent predictions (**Supplementary Table 31**). They had significantly lower CD8^+^ T−cell proportions (1.85 ± 1.03% vs. 2.12 ± 1.24%, *P* = 0.046), poorer embryo quality (52.9% vs. 76.2% with good quality AA/AB embryos, *P* = 0.015), and higher immune dysregulation scores (4.91 ± 1.33 vs. 4.41 ± 1.28, *P* = 0.042). The actual pregnancy success rate was markedly lower in discordant cases compared to consistent cases (27.9% vs. 59.5%, *P* < 0.001).

### 3.11 Post−hoc power and sample size considerations

Post−hoc power calculations, based on the observed effect sizes in the final model, indicated that the present study (n = 110) had 58% power to detect the observed difference in the primary outcome (40% vs. 60% success). For the key predictors, the study achieved 63% power for the effect of previous implantation failures (OR = 0.74) and 42% power for the CD8^+^ T−cell proportion effect (OR = 1.25) (**Supplementary Table 32**).

Sample size estimations for future validation or mechanistic studies were calculated (**Supplementary Table 33**). To achieve 80% power for detecting an effect size equivalent to that observed for CD8^+^ T−cell proportion (OR = 1.25), approximately 780 participants would be required. For a more modest effect (OR = 1.5), 210 participants would be needed, while an adequately powered validation of the primary model (AUC = 0.738) would require approximately 150 participants.

## 4 Discussion

The RIF remains a significant clinical challenge in assisted reproduction, as its occurrence cannot be fully explained by embryonic factors (61, 62). This underscores the central role of maternal endometrial receptivity (63), particularly the dysregulation of the local immune microenvironment. Although the research on endometrial immunity is advancing, current evidence often focuses on specific subsets like NK cells, yielding inconsistent conclusions and frequently failing to integrate immunological findings with the patient’s clinical history of failures (64, 65). Furthermore, the precise role and clinical significance of CD8^+^ T cells, a population critical for tissue immune surveillance and homeostasis (66), in RIF remain poorly defined. To address this, our study integrates multidimensional clinical and deep endometrial immune data through a combined ML and causal inference approach.

We identify a pivotal pathological signature in RIF defined by two counterbalancing factors: the cumulative burden of previous implantation failures and the endometrial CD8^+^ T cell proportion. This work yields a prognostic model with good discriminative performance and, more importantly, elucidates a key mechanistic pathway, mediated by CD8^+^ T cells, linking systemic immune dysregulation to pregnancy outcomes.

While embryonic aneuploidy is considered a primary cause of implantation failure, the persistence of RIF even in the context of euploid embryo transfer strongly implicates a core defect in maternal endometrial receptivity (67, 68). The endometrial immune homeostasis is fundamental to receptivity (69), and its dysregulation is regarded as a key aspect of RIF. However, previous studies have focused on isolated immune cell populations, often yielding inconsistent conclusions and lacking integration with clinical history, particularly cumulative effect of failures (70). Through systematic immune mapping and integration of clinical data, this research confirms that the number of previous implantation failures is the strongest independent negative predictor of pregnancy outcome. This aligns with recent scholarly views emphasizing that the definition of RIF should extend beyond simple counting to incorporate the concept of cumulative burden (71, 72). This dose-response relationship with the number of failures suggests that recurrent failures may not be independent events. This may reflect progressively worsening endometrial microenvironment damage, functional exhaustion of immune tolerance mechanisms (73), or the enrichment of a specific patient endotype that is refractory to conventional treatments. A key finding of this study is the defined protective role of endometrial CD8^+^ T cells.

Our data support their positive function in maintaining local homeostasis. Each 1% increase in CD8^+^ T cell proportion was associated with 25% increase in the odds of pregnancy success. Importantly, RCS analysis revealed a clear efficacy threshold at approximately 2.0%, below which the association was minimal and above which it strengthened markedly. This threshold likely represents the minimum cell density required for effective local immune surveillance, clearance of abnormal cells/tissue repair, providing a quantitative basis for future immune assessment (74, 75). Mediation analysis underscores the central mechanistic role of CD8^+^ T cells in RIF, revealing that 22.8% of the total negative effect of systemic immune dysregulation on pregnancy outcome was mediated specifically through a reduction in their endometrial proportion. This identifies CD8^+^ T cell reduction as a critical intermediary, directly linking upstream immune abnormalities to the downstream clinical outcome of implantation failure.

The protective effect of CD8^+^ T cells was notably context-dependent, showing a significant interaction with immune disorder severity. Their benefit was substantially greater in patients with severe immune dysregulation than in those with mild/moderate disorder. This suggests that in a hostile endometrial microenvironment, CD8^+^ T cells fulfill a critical compensatory role, possibly through enhanced regulation or clearance of harmful targets (76). Alternatively, it may delineate a distinct RIF endotype primarily defined by CD8^+^ T cell deficiency, which would be most amenable to targeted intervention (77, 78). This finding underscores the necessity for precise immune phenotyping to guide future therapies.

This study highlights the value of integrating machine learning with traditional statistics in reproductive medicine research (79, 80). The XGBoost outperformed logistic regression, while SHAP analysis quantified contribution of key predictors, confirming previous failure count and CD8^+^ T cell proportion as the primary drivers. DCA further showed that applying the model across clinically relevant probability thresholds yields a clear net benefit, supporting its potential as a practical decision−support tool.

This research highlights several clinically actionable insights. An endometrial CD8^+^ T cell proportion below 2.0% emerges as a potential biomarker to identify patients likely to benefit from therapies aimed at increasing or enhancing this cell population. The findings support exploring treatments targeting the CD8^+^ T cell pathway (81). Although our data suggest a benefit of immunomodulation, requires validation in prospective trials (82, 83). Integrating detailed failure history with the quantitative CD8^+^ T cell profiling is essential for advancing toward individualized RIF management.

This study identifies a high failure burden combined with low endometrial CD8^+^ T cell levels as an integrated clinical−immune signature in RIF. CD8^+^ T cells mediate impact of immune dysregulation on outcomes through a threshold−sensitive and context−dependent protective pathway. These findings advance the mechanistic understanding of RIF and provide a direct rationale for developing prognostic tools and targeted immunotherapies.

## 5 Conclusion

This study establishes that reproductive outcomes in RIF are governed by a pivotal mechanism where a high cumulative burden of previous failures and a low proportion of endometrial CD8^+^ T cells exert countervailing forces. We demonstrate that CD8^+^ T cells mediate a substantial portion of the detrimental effect of systemic immune dysregulation and exhibit a threshold−sensitive, context−dependent protective role. These findings collectively advance RIF management from a descriptive paradigm toward a mechanism−informed framework for precise prognosis and targeted immunotherapy. Prospective validation of this signature and therapeutic strategies aimed at the CD8^+^ T cell pathway are essential next steps.

## 6 Limitation

Several limitations of this study merit consideration. The retrospective, single−center design may affect generalizability, and the prospective multi−center validation is needed. Moreover, Immune profiling was performed at a single mid−luteal time point and may not capture dynamic changes during implantation window (84). Although the sample size was adequate for primary analyses, statistical power was limited for certain subgroup and interaction tests, as indicated in post−hoc analyses. Furthermore, while causal mediation and propensity−score methods were employed, residual confounding cannot be ruled out, and observed associations with treatment warrant confirmation in randomized trials (85, 86).

Notwithstanding these limitations, core finding, reproducible clinical−immune signature centered on prior failure burden and CD8^+^ T cell proportion, remain robust. These constraints highlight clear pathways for future research, particularly the need for prospective validation and interventional studies targeting the identified immune pathway.

## Data Availability

The original contributions presented in the study are included in the article/**Supplementary Material**. Further inquiries can be directed to the corresponding author.
