# Supplementary material for: Endometrial immune dysregulation shapes CD8+ T cell mediated reproductive outcomes in recurrent implantation failure: an integrated mechanistic and predictive analysis
Source: Front Immunol. 2026 Mar 30;17:1788922. doi: 10.3389/fimmu.2026.1788922 (PMC13070820; doi:10.3389/fimmu.2026.1788922)
Supplement: Supplementary file 1 [file Supplementaryfile1.zip › Table S31.docx]

**Table S31.** Characteristics of cases with discordant predictions (n = 110).

| **Characteristic** | **Consistent (n = 74)** | **Discordant (n = 36)** | ***P*-value** | **Interpretation** |
| --- | --- | --- | --- | --- |
| **Age (years)** | 33.8±3.9 | 34.1±4.4 | 0.701 | Indifference |
| **Previous failures** | 3.8±2.1 | 3.4±1.9 | 0.285 | Indifference |
| **CD8 rate (%)** | 2.12±1.24 | 1.85±1.03 | **0.046** | Inconsistent cases have lower CD8 levels |
| **Embryo quality (AA/AB)** | 76.2% | 52.9% | **0.015** | Inconsistent cases with poor embryo quality |
| **Immune disorder score** | 4.41±1.28 | 4.91±1.33 | **0.042** | Inconsistent cases with more severe immune disorders |
| **Actual Success Rate** | 59.5% | 27.9% | **< 0.001** | Consistent cases have better prognosis |
