# Supplementary material for: Endometrial immune dysregulation shapes CD8+ T cell mediated reproductive outcomes in recurrent implantation failure: an integrated mechanistic and predictive analysis
Source: Front Immunol. 2026 Mar 30;17:1788922. doi: 10.3389/fimmu.2026.1788922 (PMC13070820; doi:10.3389/fimmu.2026.1788922)
Supplement: Supplementary file 1 [file Supplementaryfile1.zip › Table S32.docx]

**Table S32.** Post-Hoc power analysis for key findings.

| **Analysis/Effect** | **Observed effect Size** | **Current sample (n = 110)** | **Required sample for 80% power** | **Achieved power** |
| --- | --- | --- | --- | --- |
| **Primary Outcome (Pregnancy Success)** | 40% vs 60%^+^ | 110 | 176 | 58% |
| **Previous failures (OR = 0.74)** | Cohen’s d=0.45 | 110 | 158 | 63% |
| **CD8 rate (OR = 1.25)** | Cohen’s d=0.32 | 110 | 242 | 42% |
| **Embryo quality (OR = 1.62)** | Cohen’s d=0.38 | 110 | 180 | 56% |
| **Interaction: CD8 rate × Immune disorder** | f² = 0.12 | 110 | 264 | 38% |
| **Model AUC = 0.738** | / | 110 | 150 | 65% |
| **PSM Treatment Effect (RD = 9%)** | Cohen’s h = 0.20 | 110 | 392 | 26% |
