# Supplementary material for: Endometrial immune dysregulation shapes CD8+ T cell mediated reproductive outcomes in recurrent implantation failure: an integrated mechanistic and predictive analysis
Source: Front Immunol. 2026 Mar 30;17:1788922. doi: 10.3389/fimmu.2026.1788922 (PMC13070820; doi:10.3389/fimmu.2026.1788922)
Supplement: Supplementary file 1 [file Supplementaryfile1.zip › Table S33.docx]

**Table S33.** Required sample sizes for different effect sizes.

| **Effect size (OR)** | **Power = 80%** | **Power = 90%** | **Current study power** |
| --- | --- | --- | --- |
| **Large (OR = 2.0)** | 88 | 118 | 94% |
| **Medium (OR = 1.5)** | 210 | 282 | 62% |
| **Small (OR = 1.25)** | 780 | 1046 | 42% |
| **CD8 rate effect (OR = 1.25)** | 780 | 1046 | 42% |
| **Previous failures (OR = 0.74)** | 620 | 832 | 51% |
