# Supplementary material for: Endometrial immune dysregulation shapes CD8+ T cell mediated reproductive outcomes in recurrent implantation failure: an integrated mechanistic and predictive analysis
Source: Front Immunol. 2026 Mar 30;17:1788922. doi: 10.3389/fimmu.2026.1788922 (PMC13070820; doi:10.3389/fimmu.2026.1788922)
Supplement: Supplementary file 1 [file Supplementaryfile1.zip › Table S1.docx]

**Table S1.** Distribution of failure outcomes and cohort composition.

| Characteristic | Number (%) |
| --- | --- |
| **Cohort composition** | |
| Met RIF criteria only | 72 (65.5) |
| Met RPL criteria only | 31 (28.2) |
| Met both RIF and RPL criteria | 7 (6.4) |
| **Failure outcomes (n = 66)** | |
| No pregnancy | 32 (48.5) |
| Biochemical pregnancy | 18 (27.3) |
| Miscarriage (< 20 weeks) | 14 (21.2) |
| Ectopic pregnancy/induced abortion | 2 (3.0) |
