# Supplementary material for: Endometrial immune dysregulation shapes CD8+ T cell mediated reproductive outcomes in recurrent implantation failure: an integrated mechanistic and predictive analysis
Source: Front Immunol. 2026 Mar 30;17:1788922. doi: 10.3389/fimmu.2026.1788922 (PMC13070820; doi:10.3389/fimmu.2026.1788922)
Supplement: Supplementary file 1 [file Supplementaryfile1.zip › Table S2.docx]

**Table S2.** Timing of immune profiling relative to clinical events.

| Timepoint | Description |
| --- | --- |
| **T0: Diagnosis** | Patients diagnosed with RIF or RPL based on clinical history (≥ 10 high-grade embryo transfers without pregnancy, or ≥ 2 losses before 20 weeks) |
| **T1: Immune sampling (baseline) ^+^** | Endometrial biopsy and peripheral blood collected during mid-luteal phase (LH+7–9 days) of a natural or hormone-replacement cycle, **before any immune-based therapy for the subsequent IVF cycle** |
| **T2: Treatment (if applicable)** | Immune-based therapy (cyclosporine, prednisone, hCG/G-CSF infusion, etc.) administered after sampling, prior to embryo transfer |
| **T3: Embryo transfer** | Embryo transfer performed in the cycle following immune sampling |
| **T4: Outcome assessment** | Pregnancy outcome (success/failure) determined by ultrasound approximately 4–5 weeks after embryo transfer |

^+^ Immune parameters measured at T1 represent baseline predictors, as they were obtained before any intervention for the index cycle and before the embryo transfer that defined the outcome.
