# Supplementary material for: Endometrial immune dysregulation shapes CD8+ T cell mediated reproductive outcomes in recurrent implantation failure: an integrated mechanistic and predictive analysis
Source: Front Immunol. 2026 Mar 30;17:1788922. doi: 10.3389/fimmu.2026.1788922 (PMC13070820; doi:10.3389/fimmu.2026.1788922)
Supplement: Supplementary file 1 [file Supplementaryfile1.zip › Table S3.docx]

**Table S3.** Algebraic transformation of clinical features of patients (n = 110).

| Features | Algebra |
| --- | --- |
| Pregnancy Success (0/1) | 1 (Success) = Single, Twin  0 (Failed) = Unpregnant, Biochemical, Missed Abortion, Ectopic Pregnancy, Spontaneous Abortion, Fetal Stoppage, Induced Abortion, Abortion |
| Age group (1/2/3) | 1 = < 35  2 = 35-40  3 = > 40 |
| CD138 Status (0/1) | 1 = Positive  0 = Negative |
| Autoimmune disease (0/1) | 1 (Positive) = Sjogren’s syndrome, Antiphospholipid syndrome, Psoriasis, Purpura  0 (Negative) = None |
| Embryo quality (0/1/2/3/4) | 1 = AA  2 = AB  3 = BB  4 = BC  0 = None |
| Immune disorder score (1/2/3/4) ^+^ | 1 = 0-3.5: Basic normal or mild disorder  2 = 3.5-5.5: Moderate immune disorder  3 = 5.5-7: Severe immune disorder  4 = > 7: Extremely severe immune disorder |
| Treatment category (1/2/3/4/5) | 1 = Immune modulators (cyclosporine, prednisone, etc.)  2 = HCG/G-CSF infusion  3 = Antibiotic treatment  4 = Combination therapy (≥ 2 main treatments)  5 = No treatment |
| Cyclosporine, Prednisone, HCG Infusion, Hydroxychloroquine, GCSF Infusion, Dexamethasone (0/1) | 1 = Therapy  0 = None |

^+^ Immune disorder score=∑ | (patient value (X_i_) - median (μ_i_))/standard deviation (σ_i_) |.
