# Supplementary material for: Endometrial immune dysregulation shapes CD8+ T cell mediated reproductive outcomes in recurrent implantation failure: an integrated mechanistic and predictive analysis
Source: Front Immunol. 2026 Mar 30;17:1788922. doi: 10.3389/fimmu.2026.1788922 (PMC13070820; doi:10.3389/fimmu.2026.1788922)
Supplement: Supplementary file 1 [file Supplementaryfile1.zip › Table S4.docx]

**Table S4.** Univariate analysis of factors associated with pregnancy success (n = 110).

| **Variable** | **Category/Unit** | **OR (95% CI)** | ***P*-value** |
| --- | --- | --- | --- |
| **Demographics** | | | |
| Age | Per year increase | 0.96 (0.90-1.02) | 0.205 |
| Age group | 2 vs 1 (35-40 vs < 35) | 0.92 (0.42-2.00) | 0.828 |
| Age group | 3 vs 1 (> 40 vs < 35) | 0.72 (0.13-4.10) | 0.711 |
| BMI | Per kg/m² increase | 0.94 (0.85-1.03) | 0.166 |
| **Infertility History** | | | |
| Duration of infertility | Per year increase | 0.95 (0.89-1.02) | 0.157 |
| Number of previous miscarriages | Per miscarriage increase | 1.09 (0.84-1.43) | 0.514 |
| Previous implantation failures | Per failure increase | **0.79 (0.66-0.94)** | **0.008** |
| Total number of failures | Per failure increase | **0.92 (0.85-0.99)** | **0.040** |
| **Immune Cell Profiles** | | | |
| CD138 status | Positive vs Negative | 4.65 (0.47-46.09) | 0.188 |
| CD138 rate | Per 1% increase | 1.00 (0.92-1.08) | 0.974 |
| CD8 rate | Per 1% increase | 1.11 (0.94-1.31) | 0.213 |
| CD56 rate (NK cells) | Per 0.01% increase | 0.99 (0.90-1.09) | 0.836 |
| NK Treg rate | Per unit increase | 1.01 (0.95-1.08) | 0.685 |
| M1 M2 rate | Per unit increase | 1.00 (0.99-1.00) | 0.749 |
| CD57 rate | Per 0.01% increase | 1.03 (0.93-1.15) | 0.577 |
| CD1a rate | Per 0.01% increase | 0.98 (0.90-1.08) | 0.713 |
| CD163 rate | Per 0.01% increase | 1.02 (0.96-1.09) | 0.479 |
| CD68 rate | Per 0.01% increase | 0.99 (0.94-1.05) | 0.771 |
| FOXP3 rate | Per 0.01% increase | 1.02 (0.96-1.08) | 0.555 |
| **Immune Dysregulation** | | | |
| Immune disorder score | Per unit increase | 0.88 (0.70-1.10) | 0.255 |
| Degree of immune disorder | 2 vs 1 (Moderate vs Mild) | 0.68 (0.31-1.50) | 0.341 |
| Degree of immune disorder | 3 vs 1 (Severe vs Mild) | 0.81 (0.25-2.58) | 0.716 |
| Degree of immune disorder | 4 vs 1 (Very severe vs Mild) | 0.61 (0.05-7.04) | 0.686 |
| **Clinical Features** | | | |
| Autoimmune disease | Present vs Absent | 3.03 (0.26-35.30) | 0.372 |
| Embryo quality | 2 vs 1 (AB vs AA) | 1.83 (0.70-4.81) | 0.220 |
| Embryo quality | 3 vs 1 (BB vs AA) | 0.53 (0.18-1.53) | 0.239 |
| Embryo quality | 4 vs 1 (BC vs AA) | 0.70 (0.15-3.33) | 0.656 |
| Embryo quality | 0 vs 1 (None vs AA) | - (No events) | - |
| **Treatment Categories** | | | |
| Treatment category | 1 vs 5 (Immune modulators vs No treatment) | 1.50 (0.26-8.61) | 0.650 |
| Treatment category | 2 vs 5 (HCG/G-CSF vs No treatment) | 0.94 (0.18-4.90) | 0.944 |
| Treatment category | 3 vs 5 (Antibiotics vs No treatment) | 1.50 (0.08-27.43) | 0.783 |
| Treatment category | 4 vs 5 (Combination vs No treatment) | 1.38 (0.30-6.42) | 0.678 |
| **Specific Medications** | | | |
| Cyclosporine | Yes vs No | 1.00 (0.28-3.61) | 1.000 |
| Prednisone | Yes vs No | 1.09 (0.45-2.64) | 0.850 |
| HCG Infusion | Yes vs No | 1.21 (0.53-2.78) | 0.655 |
| Hydroxychloroquine | Yes vs No | 1.52 (0.21-11.09) | 0.679 |
| GCSF Infusion | Yes vs No | 1.64 (0.61-4.40) | 0.329 |
| Dexamethasone | Yes vs No | 1.00 (0.16-6.23) | 1.000 |

Data are presented as odds ratios (OR) with 95% confidence intervals (CI).

Univariate logistic regression was performed for each variable separately, with pregnancy success as the outcome. Continuous variables were analyzed per unit increase; categorical variables were analyzed with the indicated reference category.
