# Supplementary material for: Endometrial immune dysregulation shapes CD8+ T cell mediated reproductive outcomes in recurrent implantation failure: an integrated mechanistic and predictive analysis
Source: Front Immunol. 2026 Mar 30;17:1788922. doi: 10.3389/fimmu.2026.1788922 (PMC13070820; doi:10.3389/fimmu.2026.1788922)
Supplement: Supplementary file 1 [file Supplementaryfile1.zip › Table S5.docx]

**Table S5.** Variables selected by LASSO regression.

| **Variable Selected** | **Coefficient** | **Standardized Coefficient** | **Selection Status** |
| --- | --- | --- | --- |
| Previous implantation failures | -0.274 | -0.411 | **Selected** |
| Total number of failures | -0.082 | -0.195 | **Selected** |
| Embryo quality | 0.189 | 0.158 | **Selected** |
| CD8 rate | 0.121 | 0.103 | **Selected** |
| BMI | -0.037 | -0.089 | **Selected** |
| **Intercept** | 0.542 | / | / |
