# Supplementary material for: Endometrial immune dysregulation shapes CD8+ T cell mediated reproductive outcomes in recurrent implantation failure: an integrated mechanistic and predictive analysis
Source: Front Immunol. 2026 Mar 30;17:1788922. doi: 10.3389/fimmu.2026.1788922 (PMC13070820; doi:10.3389/fimmu.2026.1788922)
Supplement: Supplementary file 1 [file Supplementaryfile1.zip › Table S6.docx]

**Table S6.** Multivariable logistic regression analysis for pregnancy success (n = 110).

| Variable | **Model 1: Clinical Factors** | | **Model 2: Immune Factors** | | **Model 3: Combined Model** | |
| --- | --- | --- | --- | --- | --- | --- |
|  | **aOR (95% CI)** | ***P*-value** | **aOR (95% CI)** | ***P*-value** | **aOR (95% CI)** | ***P*-value** |
| **Clinical Factors** | | | | | | |
| Age (per year) | 0.95 (0.87-1.03) | 0.241 | / | / | 0.96 (0.88-1.05) | 0.388 |
| BMI (per kg/m²) | 0.92 (0.83-1.02) | 0.124 | / | / | 0.93 (0.83-1.04) | 0.198 |
| Previous implantation failures | **0.73 (0.59-0.90)** | **0.004** | / | / | **0.75 (0.60-0.94)** | **0.012** |
| Embryo quality (AB vs AA) | 1.85 (0.66-5.19) | 0.240 | / | / | 1.92 (0.67-5.51) | 0.223 |
| **Immune Factors** | | | | | | |
| CD138 status (Positive) | / | / | 3.85 (0.35-42.7) | 0.277 | 4.12 (0.37-45.9) | 0.253 |
| CD8 rate (per 1%) | / | / | 1.18 (0.97-1.44) | 0.094 | 1.15 (0.94-1.41) | 0.172 |
| NK Treg rate (per unit) | / | / | 1.03 (0.95-1.12) | 0.439 | 1.02 (0.94-1.11) | 0.580 |
| Immune disorder score | / | / | 0.85 (0.65-1.12) | 0.251 | 0.87 (0.66-1.15) | 0.336 |
| **Treatment Factors** | | | | | | |
| Treatment category (Combination) | 1.45 (0.27-7.77) | 0.664 | 1.52 (0.28-8.18) | 0.629 | 1.48 (0.28-7.86) | 0.648 |
| **Model Statistics** | | | | | | |
| Number of variables | 4 | | 4 | | 8 | |
| AIC | 142.3 | | 149.8 | | 149.9 | |
| AUC (95% CI) | 0.68 (0.58-0.78) | | 0.62 (0.51-0.73) | | 0.71 (0.61-0.81) | |
| Hosmer-Lemeshow *P*-value | 0.452 | | 0.512 | | 0.385 | |
