# Supplementary material for: Endometrial immune dysregulation shapes CD8+ T cell mediated reproductive outcomes in recurrent implantation failure: an integrated mechanistic and predictive analysis
Source: Front Immunol. 2026 Mar 30;17:1788922. doi: 10.3389/fimmu.2026.1788922 (PMC13070820; doi:10.3389/fimmu.2026.1788922)
Supplement: Supplementary file 1 [file Supplementaryfile1.zip › Table S7.docx]

**Table S7.** Comparison of ROC curves for different prediction models.

| **Model** | **Variables Included** | **AUC (95% CI)** | **Sensitivity** | **Specificity** | **Youden’s Index** |
| --- | --- | --- | --- | --- | --- |
| **Model A: Clinical Only** | Age, BMI, Previous failures, Embryo quality | 0.685 (0.590-0.781) | 70.5% | 63.6% | 0.341 |
| **Model B: Immune Only** | CD138 status, CD8 rate, NK Treg rate, Immune score | 0.618 (0.517-0.719) | 59.1% | 65.2% | 0.243 |
| **Model C: Full Model** | All LASSO-selected variables | 0.738 (0.648-0.828) | 75.0% | 68.2% | 0.432 |
| **Model D: LASSO Model** | LASSO-selected variables (5) | 0.725 (0.635-0.815) | 72.7% | 66.7% | 0.394 |
