# Supplementary material for: Endometrial immune dysregulation shapes CD8+ T cell mediated reproductive outcomes in recurrent implantation failure: an integrated mechanistic and predictive analysis
Source: Front Immunol. 2026 Mar 30;17:1788922. doi: 10.3389/fimmu.2026.1788922 (PMC13070820; doi:10.3389/fimmu.2026.1788922)
Supplement: Supplementary file 1 [file Supplementaryfile1.zip › Table S8.docx]

**Table S8.** Sensitivity analysis.

| Analysis | N | Events | Predictor | aOR (95% CI) | *P-*value | Model AUC |
| --- | --- | --- | --- | --- | --- | --- |
| **Primary analysis (full cohort)** | 110 | 44 | Previous failures | 0.74 (0.60–0.91) | 0.004 | 0.738 |
|  |  |  | CD8^+^ T‑cell proportion | 1.25 (1.03–1.52) | 0.025 |  |
|  |  |  | Embryo quality | 1.62 (1.04–2.53) | 0.033 |  |
| **Sensitivity 1: Excluding autoimmune diseases (n = 3)** | 107 | 42 | Previous failures | 0.73 (0.59–0.90) | 0.003 | 0.741 |
|  |  |  | CD8^+^ T‑cell proportion | 1.27 (1.04–1.55) | 0.018 |  |
|  |  |  | Embryo quality | 1.61 (1.03–2.52) | 0.037 |  |
| **Sensitivity 2: Excluding age > 40 years (n = 6)** | 104 | 42 | Previous failures | 0.73 (0.59–0.90) | 0.004 | 0.736 |
|  |  |  | CD8^+^ T‑cell proportion | 1.24 (1.02–1.52) | 0.033 |  |
|  |  |  | Embryo quality | 1.65 (1.05–2.58) | 0.030 |  |
| **Sensitivity 3: CD138-negative patients only (n = 106)** | 106 | 41 | Previous failures | 0.75 (0.61–0.93) | 0.008 | 0.726 |
|  |  |  | CD8^+^ T‑cell proportion | 1.21 (0.99–1.48) | 0.064 |  |
|  |  |  | Embryo quality | 1.58 (1.00–2.48) | 0.049 |  |
