# Supplementary material for: Endometrial immune dysregulation shapes CD8+ T cell mediated reproductive outcomes in recurrent implantation failure: an integrated mechanistic and predictive analysis
Source: Front Immunol. 2026 Mar 30;17:1788922. doi: 10.3389/fimmu.2026.1788922 (PMC13070820; doi:10.3389/fimmu.2026.1788922)
Supplement: Supplementary file 1 [file Supplementaryfile1.zip › Table S9.docx]

**Table S9.** Optimal hyperparameters for XGBoost model.

| **Parameter** | **Value** |
| --- | --- |
| n estimators | 150 |
| max depth | 3 |
| learning rate | 0.05 |
| subsample | 0.8 |
| colsample bytree | 0.8 |
| gamma | 0.1 |
| min child weight | 1 |
