# Supplementary material for: Endometrial immune dysregulation shapes CD8+ T cell mediated reproductive outcomes in recurrent implantation failure: an integrated mechanistic and predictive analysis
Source: Front Immunol. 2026 Mar 30;17:1788922. doi: 10.3389/fimmu.2026.1788922 (PMC13070820; doi:10.3389/fimmu.2026.1788922)
Supplement: Supplementary file 1 [file Supplementaryfile1.zip › Table S10.docx]

**Table S10.** Feature importance in XGBoost model.

| **Feature** | **Importance Score** | **Percentage** | **Cumulative %** |
| --- | --- | --- | --- |
| Previous implantation failures | 0.384 | 38.4% | 38.4% |
| CD8 rate | 0.256 | 25.6% | 64.0% |
| Embryo quality | 0.192 | 19.2% | 83.2% |
| Total number of failures | 0.098 | 9.8% | 93.0% |
| BMI | 0.070 | 7.0% | 100.0% |
