# Supplementary material for: Endometrial immune dysregulation shapes CD8+ T cell mediated reproductive outcomes in recurrent implantation failure: an integrated mechanistic and predictive analysis
Source: Front Immunol. 2026 Mar 30;17:1788922. doi: 10.3389/fimmu.2026.1788922 (PMC13070820; doi:10.3389/fimmu.2026.1788922)
Supplement: Supplementary file 1 [file Supplementaryfile1.zip › Table S11.docx]

**Table S11.** SHAP value summary for the XGBoost model.

| Feature | SHAP value | Direction of Effect | Key Value Ranges |
| --- | --- | --- | --- |
| Previous implantation failures | 0.142 | Negative | > 4 times: Strong negative impact  1-3 times: moderate negative impact  0 times: Weak positive impact |
| CD8 rate | 0.096 | Positive | > 2.5%: Strong positive impact  1.5-2.5%: Moderate positive impact  < 1.5%: Weak negative impact |
| Embryo quality | 0.072 | Positive | AA/AB: Strong positive impact  BB: Weak positive impact  BC/None: Negative impact |
| Total number of failures | 0.035 | Negative | > 6 times: Strong negative impact  3-6 times: moderate negative impact  < 3 times: Weak impact |
| BMI | 0.021 | Negative | > 25 kg/m²: Negative impact  18.5-25 kg/m²: Weak impact  < 18.5 kg/m²: Weak negative impact |
