# Supplementary material for: Endometrial immune dysregulation shapes CD8+ T cell mediated reproductive outcomes in recurrent implantation failure: an integrated mechanistic and predictive analysis
Source: Front Immunol. 2026 Mar 30;17:1788922. doi: 10.3389/fimmu.2026.1788922 (PMC13070820; doi:10.3389/fimmu.2026.1788922)
Supplement: Supplementary file 1 [file Supplementaryfile1.zip › Table S12.docx]

**Table S12.** SHAP contribution decomposition for typical patient profiles.

| **Patient Profile** | **Predicted Probability** | **Largest Positive Contributor** | **Largest Negative Contributor** | **Net SHAP** |
| --- | --- | --- | --- | --- |
| Ideal patient | 0.82 | Embryo quality = AA (+0.28) | Previous failures = 1 (-0.12) | +0.42 |
| High risk patients | 0.18 | CD8 rate = 2.1% (+0.10) | Previous failures = 6 (-0.35) | -0.52 |
| Immune advantaged patients | 0.75 | CD8 rate = 3.2% (+0.32) | Embryo quality = BB (-0.08) | +0.21 |
| Clinical challenging patients | 0.25 | BMI = 21.5 (+0.03) | Previous failures = 5 (-0.28), Embryo quality = BC (-0.15) | -0.40 |
