# Supplementary material for: Endometrial immune dysregulation shapes CD8+ T cell mediated reproductive outcomes in recurrent implantation failure: an integrated mechanistic and predictive analysis
Source: Front Immunol. 2026 Mar 30;17:1788922. doi: 10.3389/fimmu.2026.1788922 (PMC13070820; doi:10.3389/fimmu.2026.1788922)
Supplement: Supplementary file 1 [file Supplementaryfile1.zip › Table S13.docx]

**Table S13.** SHAP interaction effects.

| **Interaction Pair** | **Interaction Strength** | **Main Pattern Observed** | **Clinical Implication** |
| --- | --- | --- | --- |
| CD8 rate × Previous failures | moderate | High CD8 rate can partially offset the impact of multiple failures | For patients with high failure rates, increasing CD8+T cells may be particularly important |
| Embryo quality × Previous failures | weak | High quality embryos have a stronger protective effect on patients with high failure rates | For patients with repeated failures, high-quality embryo selection is crucial |
| CD8 rate × BMI | weak | The protective effect of CD8 rate is more pronounced within the normal BMI range | Weight management may enhance the effect of immune regulation |
