# Supplementary material for: Endometrial immune dysregulation shapes CD8+ T cell mediated reproductive outcomes in recurrent implantation failure: an integrated mechanistic and predictive analysis
Source: Front Immunol. 2026 Mar 30;17:1788922. doi: 10.3389/fimmu.2026.1788922 (PMC13070820; doi:10.3389/fimmu.2026.1788922)
Supplement: Supplementary file 1 [file Supplementaryfile1.zip › Table S14.docx]

**Table S14.** Exploratory multi-step mediation analysis.

| **Mediation Pathway** | **Indirect Effect** | **95% CI** | ***P*-value** | **Proportion Mediated** |
| --- | --- | --- | --- | --- |
| **Immune score → CD8 rate → Outcome** | -0.042 | (-0.089, 0.005) | 0.079 | 22.8% |
| **Immune score → NK Treg rate → Outcome** | -0.023 | (-0.056, 0.010) | 0.176 | 12.5% |
| **Immune score → M1 M2 rate → Outcome** | -0.005 | (-0.020, 0.010) | 0.512 | 2.7% |
| **Immune score → CD8 rate → NK Treg rate → Outcome** | -0.008 | (-0.022, 0.006) | 0.267 | 4.3% |
| **Total Indirect Effect** | -0.078 | (-0.156, 0.000) | 0.051 | 42.4% |
