# Supplementary material for: Endometrial immune dysregulation shapes CD8+ T cell mediated reproductive outcomes in recurrent implantation failure: an integrated mechanistic and predictive analysis
Source: Front Immunol. 2026 Mar 30;17:1788922. doi: 10.3389/fimmu.2026.1788922 (PMC13070820; doi:10.3389/fimmu.2026.1788922)
Supplement: Supplementary file 1 [file Supplementaryfile1.zip › Table S15.docx]

**Table S15.** Subgroup-specific mediation analysis.

| **Subgroup** | **Total Effect** | **Direct Effect** | **Indirect Effect (CD8 rate)** | **Proportion Mediated** | ***P*-value** |
| --- | --- | --- | --- | --- | --- |
| **Age < 35 years** | -0.218 | -0.168 | -0.050 | 22.9% | 0.092 |
| **Age ≥ 35 years** | -0.142 | -0.102 | -0.040 | 28.2% | 0.112 |
| **CD138 Negative** | -0.176 | -0.136 | -0.040 | 22.7% | 0.095 |
| **Severe Immune Disorder** | -0.324 | -0.198 | -0.126* | 38.9% | **0.038** |
| **Mild/Moderate Disorder** | -0.152 | -0.128 | -0.024 | 15.8% | 0.231 |
