# Supplementary material for: Endometrial immune dysregulation shapes CD8+ T cell mediated reproductive outcomes in recurrent implantation failure: an integrated mechanistic and predictive analysis
Source: Front Immunol. 2026 Mar 30;17:1788922. doi: 10.3389/fimmu.2026.1788922 (PMC13070820; doi:10.3389/fimmu.2026.1788922)
Supplement: Supplementary file 1 [file Supplementaryfile1.zip › Table S16.docx]

**Table S16.** Multidimensional stratified subgroup analysis (n = 110).

| Variable | Age < 35 years (n = 66) | | Age ≥ 35 years (n = 44) | | *P*-interaction |
| --- | --- | --- | --- | --- | --- |
|  | **aOR (95% CI)** | ***P*-value** | **aOR (95% CI)** | ***P*-value** |  |
| Previous implantation failures | 0.68 (0.51-0.91) | **0.010** | 0.81 (0.60-1.09) | 0.162 | 0.228 |
| CD8 rate | 1.28 (1.01-1.62) | **0.044** | 1.22 (0.95-1.57) | 0.122 | 0.689 |
| Embryo quality | 1.85 (1.08-3.17) | **0.025** | 1.42 (0.78-2.59) | 0.253 | 0.421 |
| Total number of failures | 0.91 (0.81-1.02) | 0.099 | 0.97 (0.86-1.09) | 0.590 | 0.336 |
| BMI | 0.96 (0.84-1.10) | 0.553 | 0.92 (0.79-1.07) | 0.266 | 0.674 |
| Model AUC | 0.781 | | 0.689 | | / |
| Events/Sample | 27/66 (40.9%) | | 17/44 (38.6%) | | / |
