# Supplementary material for: Endometrial immune dysregulation shapes CD8+ T cell mediated reproductive outcomes in recurrent implantation failure: an integrated mechanistic and predictive analysis
Source: Front Immunol. 2026 Mar 30;17:1788922. doi: 10.3389/fimmu.2026.1788922 (PMC13070820; doi:10.3389/fimmu.2026.1788922)
Supplement: Supplementary file 1 [file Supplementaryfile1.zip › Table S17.docx]

**Table S17.** Multivariable analysis stratified by CD138 status (n = 110).

| Variable | CD138 Negative (n = 106) | | CD138 Positive (n = 4) | | *P*-interaction |
| --- | --- | --- | --- | --- | --- |
|  | **aOR (95% CI)** | ***P*-value** | **aOR (95% CI)** | ***P*-value** |  |
| Previous implantation failures | 0.75 (0.61-0.93) | **0.009** | 0.41 (0.08-2.08) | 0.280 | 0.391 |
| CD8 rate | 1.24 (1.02-1.51) | **0.034** | 2.85 (0.42-19.36) | 0.284 | 0.288 |
| Embryo quality | 1.61 (1.03-2.51) | **0.035** | 3.21 (0.23-45.17) | 0.385 | 0.620 |
| Total number of failures | 0.94 (0.86-1.03) | 0.175 | 0.72 (0.35-1.50) | 0.380 | 0.322 |
| BMI | 0.94 (0.84-1.05) | 0.263 | 0.62 (0.25-1.54) | 0.301 | 0.334 |
| Model AUC | 0.738 | | 0.952 | | / |
| Events/Sample | 41/106 (38.7%) | | 3/4 (75.0%) | | / |
