# Supplementary material for: Endometrial immune dysregulation shapes CD8+ T cell mediated reproductive outcomes in recurrent implantation failure: an integrated mechanistic and predictive analysis
Source: Front Immunol. 2026 Mar 30;17:1788922. doi: 10.3389/fimmu.2026.1788922 (PMC13070820; doi:10.3389/fimmu.2026.1788922)
Supplement: Supplementary file 1 [file Supplementaryfile1.zip › Table S18.docx]

**Table S18.** Multivariable analysis stratified by degree of immune disorder (n = 110).

| Variable | **Mild/Moderate (n = 92)** | | **Severe/Very Severe (n = 18)** | | *P*-interaction |
| --- | --- | --- | --- | --- | --- |
|  | **aOR (95% CI)** | ***P*-value** | **aOR (95% CI)** | ***P*-value** |  |
| Previous implantation failures | 0.77 (0.61-0.97) | **0.028** | 0.65 (0.40-1.05) | 0.078 | 0.537 |
| CD8 rate | 1.18 (0.96-1.45) | 0.118 | **1.52 (1.08-2.14)** | **0.017** | **0.034** |
| Embryo quality | 1.55 (0.98-2.46) | 0.062 | 2.01 (0.78-5.20) | 0.151 | 0.582 |
| Total number of failures | 0.94 (0.86-1.03) | 0.194 | 0.92 (0.74-1.15) | 0.470 | 0.862 |
| BMI | 0.94 (0.84-1.06) | 0.307 | 0.93 (0.68-1.27) | 0.646 | 0.950 |
| Model AUC | 0.712 | | 0.865 | | / |
| Events/Sample | 37/92 (40.2%) | | 7/18 (38.9%) | | / |
