# Supplementary material for: Endometrial immune dysregulation shapes CD8+ T cell mediated reproductive outcomes in recurrent implantation failure: an integrated mechanistic and predictive analysis
Source: Front Immunol. 2026 Mar 30;17:1788922. doi: 10.3389/fimmu.2026.1788922 (PMC13070820; doi:10.3389/fimmu.2026.1788922)
Supplement: Supplementary file 1 [file Supplementaryfile1.zip › Table S19.docx]

**Table S19.** Multivariable analysis stratified by embryo quality (n = 110).

| Variable | **Good Quality (AA/AB, n = 67)** | | **Poor Quality (BB/BC/None, n = 43)** | | *P*-interaction |
| --- | --- | --- | --- | --- | --- |
|  | **aOR (95% CI)** | ***P*-value** | **aOR (95% CI)** | ***P*-value** |  |
| Previous implantation failures | 0.71 (0.55-0.92) | **0.009** | 0.78 (0.57-1.07) | 0.123 | 0.602 |
| CD8 rate | 1.22 (0.97-1.53) | 0.091 | 1.29 (0.97-1.72) | 0.082 | 0.775 |
| Embryo quality | / | / | / | / | /- |
| Total number of failures | 0.93 (0.84-1.03) | 0.164 | 0.95 (0.83-1.09) | 0.462 | 0.801 |
| BMI | 0.91 (0.79-1.05) | 0.185 | 0.98 (0.83-1.16) | 0.822 | 0.520 |
| Model AUC | 0.752 | | 0.718 | | / |
| Events/Sample | 32/67 (47.8%) | | 12/43 (27.9%) | | / |
