# Supplementary material for: Endometrial immune dysregulation shapes CD8+ T cell mediated reproductive outcomes in recurrent implantation failure: an integrated mechanistic and predictive analysis
Source: Front Immunol. 2026 Mar 30;17:1788922. doi: 10.3389/fimmu.2026.1788922 (PMC13070820; doi:10.3389/fimmu.2026.1788922)
Supplement: Supplementary file 1 [file Supplementaryfile1.zip › Table S20.docx]

**Table S20.** Multivariable analysis stratified by treatment strategy (n = 110).

| Variable | **Immune-Based Therapy (n = 64)** | | **Other Therapies (n = 46)** | | *P*-interaction |
| --- | --- | --- | --- | --- | --- |
|  | **aOR (95% CI)** | ***P*-value** | **aOR (95% CI)** | ***P*-value** |  |
| Previous implantation failures | 0.72 (0.56-0.93) | **0.012** | 0.77 (0.57-1.04) | 0.086 | 0.732 |
| CD8 rate | 1.32 (1.05-1.66) | **0.017** | 1.15 (0.88-1.50) | 0.305 | 0.338 |
| Embryo quality | 1.75 (1.05-2.92) | **0.032** | 1.48 (0.82-2.67) | 0.196 | 0.641 |
| Total number of failures | 0.92 (0.83-1.02) | 0.108 | 0.96 (0.85-1.09) | 0.555 | 0.621 |
| BMI | 0.92 (0.80-1.06) | 0.250 | 0.97 (0.83-1.14) | 0.717 | 0.679 |
| Model AUC | 0.768 | | 0.703 | | / |
| Events/Sample | 28/64 (43.8%) | | 16/46 (34.8%) | | / |
