# Supplementary material for: Endometrial immune dysregulation shapes CD8+ T cell mediated reproductive outcomes in recurrent implantation failure: an integrated mechanistic and predictive analysis
Source: Front Immunol. 2026 Mar 30;17:1788922. doi: 10.3389/fimmu.2026.1788922 (PMC13070820; doi:10.3389/fimmu.2026.1788922)
Supplement: Supplementary file 1 [file Supplementaryfile1.zip › Table S21.docx]

**Table S21.** RCS segmentation effect estimation.

|  | **OR (95% CI)** | ***P*-value** | **Slope** | **Clinical Interpretation** |
| --- | --- | --- | --- | --- |
| **RCS Piecewise Effect Estimates for CD8 rate [CD8 rate Range (%) ]** | | | | |
| **< 1.2%** | 0.85 (0.65-1.11) | 0.234 | -0.163 | No significant impact on pregnancy outcomes |
| **1.2-1.9%** | 1.18 (0.94-1.48) | 0.156 | +0.165 | Trend positive impact |
| **1.9-2.6%** | **1.52 (1.15-2.01)** | **0.003** | +0.419 | Significant positive impact |
| **> 2.6%** | **1.83 (1.28-2.62)** | **0.001** | +0.604 | Strong positive impact |
| **Overall trend** | / | **0.025** | +0.257 | Significant positive linear trend |
| **RCS Piecewise Effect Estimates for Previous failures [Previous failures Range]** | | | | |
| **0-1 failures** | 1.12 (0.86-1.46) | 0.398 | +0.113 | No significant impact |
| **2-3 failures** | **0.68 (0.52-0.89)** | **0.005** | -0.386 | Significant negative impact |
| **4-5** **failures** | **0.49 (0.34-0.71)** | **< 0.001** | -0.713 | Strong negative impact |
| **> 5 failures** | 0.42 (0.26-0.68) | **< 0.001** | -0.868 | Extremely strong negative impact |
| **Overall trend** | / | **0.008** | -0.296 | Significant negative linear trend |
