# Supplementary material for: Endometrial immune dysregulation shapes CD8+ T cell mediated reproductive outcomes in recurrent implantation failure: an integrated mechanistic and predictive analysis
Source: Front Immunol. 2026 Mar 30;17:1788922. doi: 10.3389/fimmu.2026.1788922 (PMC13070820; doi:10.3389/fimmu.2026.1788922)
Supplement: Supplementary file 1 [file Supplementaryfile1.zip › Table S22.docx]

**Table S22.** Potential threshold effects detected by RCS analysis.

| **Variable** | **Potential threshold** | **Below threshold** | **Above threshold** | **Difference** | ***P*-value** |
| --- | --- | --- | --- | --- | --- |
| **CD8 rate** | 2.0% | OR = 1.08 (0.86-1.36) | OR = 1.52 (1.18-1.96) | +40.7% | **0.024** |
| **Previous failures** | 3 failures | OR = 0.92 (0.74-1.14) | OR = 0.58 (0.43-0.78) | -37.0% | **0.011** |
| **Age** | 37 years | OR = 1.02 (0.94-1.11) | OR = 0.86 (0.72-1.03) | -15.7% | 0.098 |
| **Immune score** | 5.5 points | OR = 0.94 (0.78-1.13) | OR = 0.82 (0.63-1.07) | -12.8% | 0.382 |
| **NK/Treg rate** | 8.0 | OR = 1.01 (0.95-1.07) | OR = 1.03 (0.94-1.13) | +2.0% | 0.795 |
