# Supplementary material for: Endometrial immune dysregulation shapes CD8+ T cell mediated reproductive outcomes in recurrent implantation failure: an integrated mechanistic and predictive analysis
Source: Front Immunol. 2026 Mar 30;17:1788922. doi: 10.3389/fimmu.2026.1788922 (PMC13070820; doi:10.3389/fimmu.2026.1788922)
Supplement: Supplementary file 1 [file Supplementaryfile1.zip › Table S23.docx]

**Table S23.** Bootstrap internal validation of model performance.

| **Model** | **Original AUC** | **Bootstrap optimism** | **Optimism-corrected AUC** | **95% CI of Corrected AUC** | **Shrinkage factor** |
| --- | --- | --- | --- | --- | --- |
| **Clinical Model** | 0.685 | 0.032 | 0.653 | (0.581-0.725) | 0.89 |
| **Immune Model** | 0.618 | 0.045 | 0.573 | (0.497-0.649) | 0.84 |
| **Combined Model** | 0.738 | 0.028 | 0.710 | (0.642-0.778) | 0.91 |
| **XGBoost Model** | 0.762 | 0.035 | 0.727 | (0.660-0.794) | 0.88 |
| **Final LASSO Model** | 0.725 | 0.026 | 0.699 | (0.630-0.768) | 0.92 |
