# Supplementary material for: Endometrial immune dysregulation shapes CD8+ T cell mediated reproductive outcomes in recurrent implantation failure: an integrated mechanistic and predictive analysis
Source: Front Immunol. 2026 Mar 30;17:1788922. doi: 10.3389/fimmu.2026.1788922 (PMC13070820; doi:10.3389/fimmu.2026.1788922)
Supplement: Supplementary file 1 [file Supplementaryfile1.zip › Table S24.docx]

**Table S24.** Calibration performance from Bootstrap validation.

| **Metric** | **Clinical Model** | **Immune Model** | **Combined Model** | **XGBoost Model** | **Final LASSO Model** |
| --- | --- | --- | --- | --- | --- |
| **Calibration-in-the-large** | 0.02 | 0.05 | 0.01 | 0.03 | 0.01 |
| **Calibration Slope** | 0.89 | 0.84 | 0.91 | 0.88 | 0.92 |
| **Harrell’s E90** | 0.87 | 0.82 | 0.90 | 0.86 | 0.91 |
| **Brier Score (original)** | 0.208 | 0.218 | 0.195 | 0.189 | 0.201 |
| **Brier Score (corrected)** | 0.221 | 0.235 | 0.210 | 0.203 | 0.214 |
| **Expected/Observed Ratio** | 0.98 | 0.95 | 1.02 | 0.97 | 1.01 |
