# Supplementary material for: Endometrial immune dysregulation shapes CD8+ T cell mediated reproductive outcomes in recurrent implantation failure: an integrated mechanistic and predictive analysis
Source: Front Immunol. 2026 Mar 30;17:1788922. doi: 10.3389/fimmu.2026.1788922 (PMC13070820; doi:10.3389/fimmu.2026.1788922)
Supplement: Supplementary file 1 [file Supplementaryfile1.zip › Table S25.docx]

**Table S25.** Comparison of DCA across different prediction models.

| Threshold Range | Clinical Model | Immune Model | Combined Model | XGBoost Model | Optimal Model |
| --- | --- | --- | --- | --- | --- |
| 20-40% (Main clinical scope) | 0.23 | 0.18 | 0.27 | **0.30** | XGBoost |
| 40-60% (Individualized decision-making) | 0.14 | 0.11 | 0.18 | **0.21** | XGBoost |
| Overall net benefit (0-100%) | 0.16 | 0.12 | 0.19 | **0.22** | XGBoost |
| Standardized net benefit | 0.10 | 0.07 | 0.12 | **0.14** | XGBoost |
