# Supplementary material for: Endometrial immune dysregulation shapes CD8+ T cell mediated reproductive outcomes in recurrent implantation failure: an integrated mechanistic and predictive analysis
Source: Front Immunol. 2026 Mar 30;17:1788922. doi: 10.3389/fimmu.2026.1788922 (PMC13070820; doi:10.3389/fimmu.2026.1788922)
Supplement: Supplementary file 1 [file Supplementaryfile1.zip › Table S26.docx]

**Table S26.** Sensitivity analysis for PSM.

| **Sensitivity Parameter (Γ)** | ***P*-value Range** | **Significant?** | **Interpretation** |
| --- | --- | --- | --- |
| 1.0 (baseline) | 0.219 | No | Results when unobserved confounding effects can be ignored |
| 1.5 | 0.219-0.417 | No | A small amount of unobserved confounding does not affect the conclusion |
| 2.0 | 0.219-0.576 | No | Moderate unobserved confounding does not affect the conclusion |
| 2.5 | 0.219-0.694 | No | Large unobserved confounding does not affect the conclusion |
| E-value | 1.98 | / | An unobserved confounding with OR = 1.98 is required to explain the observed effects |
