# Supplementary material for: Endometrial immune dysregulation shapes CD8+ T cell mediated reproductive outcomes in recurrent implantation failure: an integrated mechanistic and predictive analysis
Source: Front Immunol. 2026 Mar 30;17:1788922. doi: 10.3389/fimmu.2026.1788922 (PMC13070820; doi:10.3389/fimmu.2026.1788922)
Supplement: Supplementary file 1 [file Supplementaryfile1.zip › Table S27.docx]

**Table S27.** Baseline characteristics before and after PSM (n = 110).

| **Variable** | **Before Matching** | **After Matching** | **Standardization differences** | **Balance improvement** | Before matching | Match after |
| --- | --- | --- | --- | --- | --- | --- |
|  | Treatment (n = 64) | Control (n = 46) | Treatment (n = 40) | Control (n = 40) |  |  |
| **Age (years)** | 34.2±4.1 | 33.7±4.4 | 33.9±4.0 | 34.1±4.2 | 0.12 | 0.05 |
| **BMI (kg/m²)** | 22.3±3.2 | 22.6±3.5 | 22.4±3.1 | 22.5±3.3 | 0.09 | 0.03 |
| **Previous failures** | 3.4±1.8 | 3.8±2.1 | 3.5±1.7 | 3.6±1.9 | 0.20 | 0.05 |
| **Total failures** | 4.7±2.9 | 5.2±3.2 | 4.8±2.7 | 4.9±2.8 | 0.16 | 0.04 |
| **Embryo quality (AA/AB)** | 65.6% | 58.7% | 67.5% | 65.0% | 0.14 | 0.05 |
| **CD138 positive** | 3.1% | 4.3% | 2.5% | 2.5% | 0.06 | 0.00 |
| **CD8 rate (%)** | 2.01±1.12 | 1.89±1.08 | 2.03±1.10 | 1.98±1.05 | 0.11 | 0.05 |
| **Immune score** | 4.68±1.30 | 4.80±1.35 | 4.71±1.28 | 4.73±1.30 | 0.09 | 0.02 |
| **Mean propensity score** | 0.58±0.21 | 0.42±0.23 | 0.55±0.18 | 0.54±0.19 | 0.73 | 0.05 |
