# Supplementary material for: Endometrial immune dysregulation shapes CD8+ T cell mediated reproductive outcomes in recurrent implantation failure: an integrated mechanistic and predictive analysis
Source: Front Immunol. 2026 Mar 30;17:1788922. doi: 10.3389/fimmu.2026.1788922 (PMC13070820; doi:10.3389/fimmu.2026.1788922)
Supplement: Supplementary file 1 [file Supplementaryfile1.zip › Table S28.docx]

**Table S28.** Treatment effect estimates after PSM (n = 110).

| **Analysis method** | **Samples** | **Treatment** | **Control** | **Risk difference** | **OR (95% CI)** | ***P*-value** |
| --- | --- | --- | --- | --- | --- | --- |
| **Before Matching** | 110 | 43.8% (28/64) | 34.8% (16/46) | +9.0% (-7.8% to +25.8%) | 1.46 (0.70-3.03) | 0.315 |
| **1:1 Nearest Neighbor** | 80 | 45.0% (18/40) | 32.5% (13/40) | +12.5% (-7.0% to +32.0%) | 1.70 (0.73-3.95) | 0.219 |
| **1:2 Caliper Matching** | 102 | 44.2% (27/61) | 34.1% (14/41) | +10.1% (-6.8% to +27.0%) | 1.53 (0.71-3.30) | 0.282 |
| **IPTW (Inverse Probability)** | 110 | 43.8% | 34.8% | +9.0% (-7.8% to +25.8%) | 1.46 (0.70-3.03) | 0.315 |
| **Stratification (5 strata)** | 110 | 43.8% | 34.8% | +9.0% (-6.5% to +24.5%) | 1.46 (0.71-3.01) | 0.307 |
