# Supplementary material for: Endometrial immune dysregulation shapes CD8+ T cell mediated reproductive outcomes in recurrent implantation failure: an integrated mechanistic and predictive analysis
Source: Front Immunol. 2026 Mar 30;17:1788922. doi: 10.3389/fimmu.2026.1788922 (PMC13070820; doi:10.3389/fimmu.2026.1788922)
Supplement: Supplementary file 1 [file Supplementaryfile1.zip › Table S29.docx]

**Table S29.** Multi-model prediction consistency analysis.

| **Patient stratum** | **Number of patients** | **Models agreeing on success** | **Models agreeing on failure** | **Actual success rate** |
| --- | --- | --- | --- | --- |
| **Unanimous Success** | 18 | 5/5 models | 0/5 models | 83.3% (15/18) |
| **Majority Success (≥ 3 models)** | 32 | ≥ 3 models | ≤ 2 models | 62.5% (20/32) |
| **Divided Prediction** | 36 | 2-3 models | 2-3 models | 41.7% (15/36) |
| **Majority Failure (≥ 3 models)** | 19 | ≤ 2 models | ≥ 3 models | 21.1% (4/19) |
| **Unanimous Failure** | 5 | 0/5 models | 5/5 models | 0.0% (0/5) |
| **Overall** | 110 | / | / | 40.0% |
