# Supplementary material for: Endometrial immune dysregulation shapes CD8+ T cell mediated reproductive outcomes in recurrent implantation failure: an integrated mechanistic and predictive analysis
Source: Front Immunol. 2026 Mar 30;17:1788922. doi: 10.3389/fimmu.2026.1788922 (PMC13070820; doi:10.3389/fimmu.2026.1788922)
Supplement: Supplementary file 1 [file Supplementaryfile1.zip › Table S30.docx]

**Table S30.** Model agreement statistics.

| **Agreement metric** | **Value** | **95% CI** | **Interpretation** |
| --- | --- | --- | --- |
| **Overall Agreement (Kappa)** | 0.48 | (0.39-0.57) | Moderate consistency |
| **Pairwise Agreement** | 72.3% | (68.1%-76.5%) | Good consistency |
| **Fleiss’ Kappa** | 0.42 | (0.35-0.49) | Moderate consistency |
| **Intraclass Correlation (ICC)** | 0.65 | (0.57-0.72) | Good reliability |
| **Consensus Strength** | 0.71 | (0.64-0.78) | stronger |
| **Predictive Certainty Index** | 0.58 | (0.50-0.66) | Moderate certainty |
